# An Ultrafast Charge‐Driven Topological Intercalation Prelithiation Strategy for Carbon‐Silicon Composite Anodes

**DOI:** 10.1002/advs.202506636

**Published:** 2025-06-04

**Authors:** Yifan Zhao, Liang Zhang, Qian Liu, Mingyu Liu, Jiajia Shen, Hui Ma, Juejing Dai, Xi Yu, Jianhua Yan

**Affiliations:** ^1^ College of Textiles Donghua University Shanghai 201620 China; ^2^ College of Material and Textile Engineering Jiaxing University Zhejiang 314001 China; ^3^ School of Textile Materials and Engineering Wuyi University Jiangmen 529020 China

**Keywords:** carbon silicon nanofiber anode, charge‐driven prelithiation model, initial coulombic efficiency, stable solid electrolyte interface, topological orientation intercalation

## Abstract

Prelithiation emerges as an effective technique to enhance the initial Coulombic efficiency (ICE) and cycling stability of silicon oxide based carbon composite (C/SiO_x_) anodes, yet traditional approaches remain plagued by sluggish kinetics, cumbersome procedures, safety hazards, and inadequate precision. Here, a facile topological intercalation prelithiation method capable of forming a robust and homogeneous solid electrolyte interface (SEI) network on porous C/SiO_x_ nanofiber anodes in merely 30 s is reported. Through constructing three charge‐driven topology models derived from flexible SiO_x_/porous carbon nanofiber (SiO_x_/PCNF) films, the mechanism of this fast Li^+^‐intercalation process is unraveled. Abundant surface defects on SiO_x_/PCNF enhance lithium salt adsorption and dissociation, while the active solvated Li^+^‐ions can quickly intercalate into SiO_x_/PCNF along an orientation pathway, realizing a high ICE of 99.44%. This topological prelithiation forges a 3D inorganic‐rich SEI architecture that dualizes functionality: It curtails electrolyte degradation while alleviating volume fluctuation and mechanical stress, while enabling precision Li^+^‐ion replenishment. This topochemical paradigm not only achieves ICE reinforcement and cycling resilience (1000 stable cycles), but also slashes prelithiation duration by orders of magnitude.

## Graphical abstract

In this work, an ultrafast topological intercalation prelithiation method capable of forming a uniformly 3D stable solid electrolyte interface (SEI) network on porous C/SiO_x_ nanofiber anodes within mere 30 s is designed. The SEI significantly enhances the initial Coulombic efficiency of the anodes to 99.44%, and the corresponding batteries show a stable cycling over 1000 times.

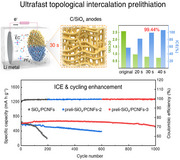

## 1 Introduction

The energy density of lithium‐ion batteries (LIBs) utilizing graphite anodes is nearing its theoretical ceiling, constrained fundamentally by graphite's inherent Li^+^ storage capacity.^[^ ^1^, ^2^ ^]^ In this landscape, C/SiO_x_ composite anodes have emerged as promising candidates for powering next‐generation high‐energy LIBs, boasting superior specific capacity, favorable operating voltage, and cost‐effective manufacturability.^[^ ^3^, ^4^ ^]^ Yet SiO_x_ anodes face critical challenges: irreversible Li^+^ consumption during initial SEI formation slashes their ICE below 80%, while severe volume expansion‐contraction cycles trigger active material degradation, perpetuating SEI reorganization and depleting Li‐ion reserves – a dual assault on cycling performance.^[^ ^5^, ^6^, ^7^ ^]^ Electrochemically derived from electrolyte reduction at low anode potentials, the SEI forms a vital regulatory interface governing Li^+^ shuttling between electrodes and electrolyte. Its structural integrity and compositional uniformity thus become decisive factors in battery longevity. Prelithiation emerges as an effective strategy to counteract significant Li^+^ loss during electrochemical cycling, stabilize the SEI, and enhance ICE and cycling stability.^[^ ^8^ ^]^ By engineering Li^+^ reservoirs within the anode matrix, this technique directly addresses the root causes of performance decay, charting a path toward robust energy storage solutions.^[^ ^9^, ^10^ ^]^

Common prelithiation methods include physical contact, chemical, and electrochemical approaches.^[^ ^11^, ^12^, ^13^, ^14^, ^15^, ^16^, ^17^, ^18^ ^]^ The physical contact method employs direct pressure‐driven interaction between the anode and lithium foil or molten lithium, enabling Li^+^ insertion. While simple, eco‐friendly, and compatible with existing battery production, this method suffers from suboptimal lithium‐source‐anode interface contact, electronic channel instability, and excessive inactive lithium generation.^[^ ^19^ ^]^ In‐situ prelithiation further risks triggering parasitic side reactions.^[^ ^20^ ^]^ Chemical prelithiation immerses the anode in active lithium compound solutions, leveraging redox potential disparities for controlled lithiation. Though efficient and safe, the higher redox potential of typical solutions relative to carbon anodes risks inert reactivity and microstructural compromise. Electrochemical prelithiation, on the other hand, has garnered widespread attention due to its controllability. It involves establishing electrochemical pathways between lithium foil, organic electrolyte, and the anode, along with lithium insertion driven by an electric field. Nonetheless, electrochemical prelithiation often entails the disassembly and reassembly of the battery, which is both laborious and inefficient.^[^ ^21^ ^]^ Furthermore, most studies concentrate on prelithiation performance, such as achieving high ICE, while neglecting the underlying mechanisms. The connection between the decomposition kinetics of prelithiation solutions and the SEI formation process remains an area to be explored.^[^ ^22^ ^]^

In this work, we developed three innovative topochemical prelithiation models using flexible topological SiO_x_/PCNF films: slow spontaneous, moderate electrically driven, and ultrafast electrically driven, resulting in in‐situ engineered structurally distinct SEI layers.^[^ ^23^, ^24^ ^]^ Through time‐of‐flight secondary ion mass spectrometry (ToF‐SIMS), X‐ray photoelectron spectroscopy (XPS), and electrochemical impedance spectroscopy (EIS) analyses, we unraveled SEI property variations across models and the stability mechanisms of intercalated SEI architectures. Building on these insights, we devised an efficient topochemical prelithiation strategy: immersing SiO_x_/PCNF in 1 M LiPF₆ solution and connecting them to Li‐metal sheets via external circuits. The LiPF₆ solution's ion pair distribution‐dominated by contact ion pairs (CIP) and solvent‐separated ion pairs (SSIP) with low pairing energy‐enabled the formation of a stable, inorganic‐rich SEI (LiF, Li₂CO₃, Li₂O), effectively curbing electrolyte decomposition side reactions. Simultaneously, the self‐supporting nanofiber film's mesoporous nanostructure shortened Li^+^ diffusion paths, mitigated pore network defect impacts, and provided abundant diffusion channels. Coupled with high specific surface area (SSA) and uniform pore distribution, this strategy established a 3D Li‐ion‐saturated conductive network, suppressing SiO_x_/C anode volume expansion. Achieving 99.44% ICE and 1000‐cycle robustness in just 30 s, this approach holds promise for scalable roll‐to‐roll electrode fabrication.

## 2 Results

### 2.1 Design of Topochemical Prelithiation Models and Ultrafast Prelithiation Mechanism to Generate Dense and Stable 3D Inorganic‐Rich SEI

**Figure 1:**
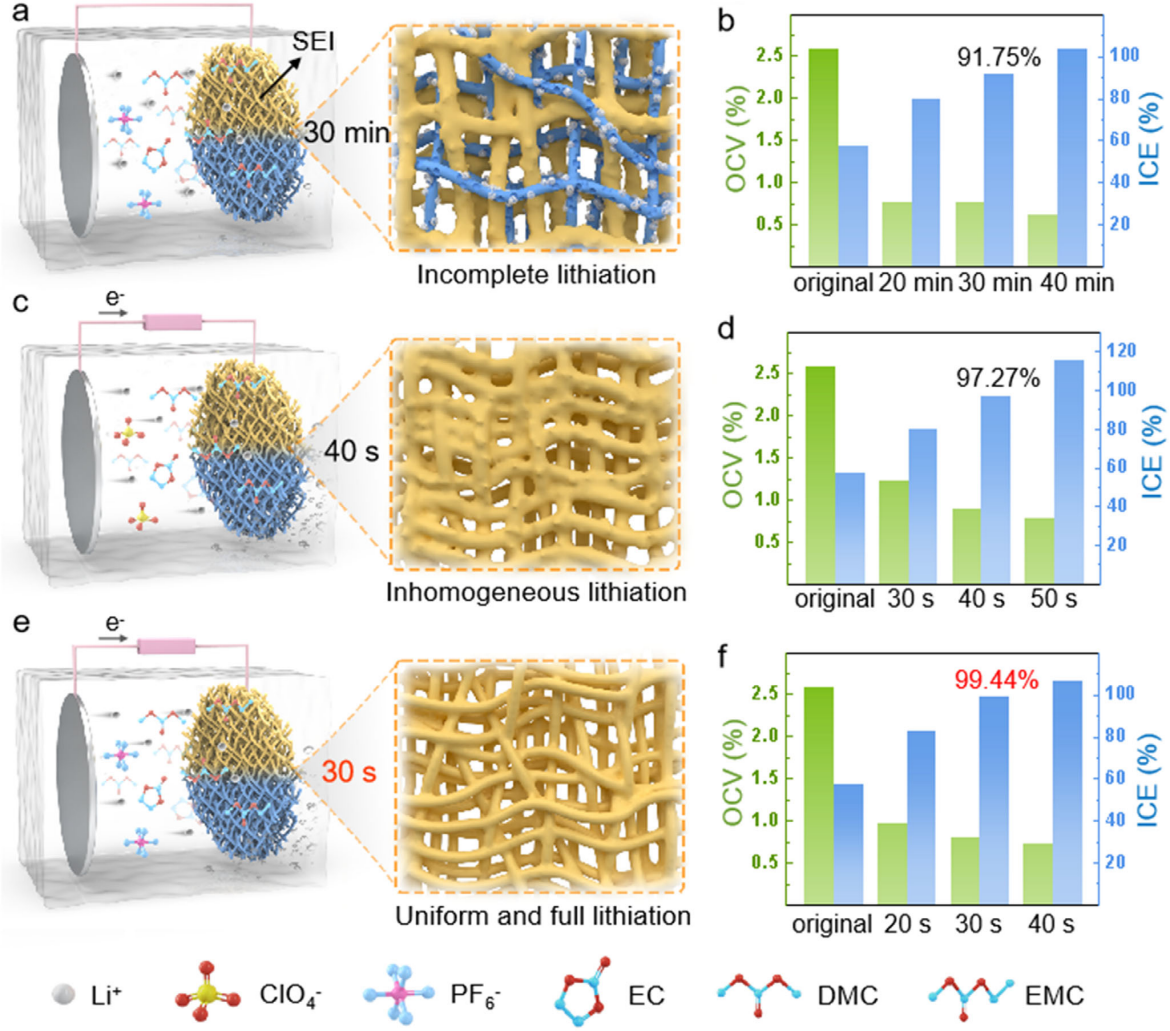
Model designs for topological intercalation prelithiation and mechanism analysis. a,b) Model 1 (slow spontaneous prelithiation model) and the corresponding ICE values over time. c,d) Model 2 (moderate electrically‐driven prelithiation model) and the corresponding ICE values over time. e,f) Model 3 (ultrafast electrically driven prelithiation model) and the corresponding ICE values over time.

**Figure** **1** shows a schematic diagram of Li^+^ intercalation into topological SiO_x_/PCNF architectures and the formation of brown SEIs through three distinct topochemical prelithiation models. The first model (Figure 1a) features a thin SiO_x_/PCNF anode coupled with a Li‐metal sheet via conductive wiring, immersed in 1 M LiPF_6_ electrolyte (within an ethylene carbonate (EC): dimethyl carbonate (DMC): methyl ethyl carbonate (EMC) solvent blend at a 1:1:1 volume ratio). The fibrous SiO_x_/PCNF matrix offered efficient electron‐transport pathways with minimized activation energy, ensuring robust electron availability for sustained lithiation. Termed a slow spontaneous lithiation process, Model 1 depended entirely on Li^+^ diffusion kinetics. While Li^+^ was abundant in the electrolyte, their migration within localized regions faced constraints from diffusion‐path selectivity and fluctuating Li^+^ concentrations. Li^+^ preferentially migrated toward active sites ‐such as Si‐O and C = C bonds – via shorter conductive routes, while alternative pathways exhibited elevated diffusion resistance and Li^+^ depletion, leaving NFs unaltered. As electron conduction vastly outpaced Li^+^ mobility, brown SEIs formed vertically along the dominant electron‐transfer trajectories. Experimental outcomes (Figure 1b) reveal that this model demanded 30 min to elevate the ICE to 91.75%, with the prelithiated anode (marked as SiO_x_/PCNF‐1) retaining partially lithiated domains.

To achieve an efficient lithiation process, two novel models were strategically designed. Both models employed an external electric field to accelerate Li^+^ and electron migration. The second model utilized 1 M LiClO_4_ dissolved in EC: DMC: EMC (1:1:1 volume ratio), while the third model maintained LiPF_6_ as the electrolyte. Owing to LiClO_4_’s more stable solvation structure and sluggish desolvation kinetics, Li^+^ preferentially migrated through low‐resistance pathways toward the NF surface, creating uneven ion distribution (Figure 1c). Concurrently, the SEI layer formed during LiClO_4_ decomposition contained abundant organic components like alkyl lithium carbonate,^[^ ^25^ ^]^ which remarkably infiltrated inter‐NF pores in localized regions, obstructing homogeneous Li^+^ transport and insertion. These synergistic effects culminated in heterogeneous lithiation reactions across the anode (SiO_x_/PCNF‐2) surface. Despite achieving 97.27% ICE within merely 40 s (Figure 1d), an elevated voltage of 8 V became necessary to overcome substantial interfacial resistance. This methodology was designated as the moderate electrically‐driven prelithiation model.

In stark contrast, the lithiation reaction in Model 3 unfolded with strikingly more efficient and homogeneous distribution (Figure 1e). Crucially, the LiPF₆ prelithiation solution exhibited notably lower pairing energy, enabling Li⁺ to exist as CIPs (contact ion pairs) and SSIPs (solvent‐separated ion pairs), as supported by prior studies on the ion pairing dynamics in carbonate‐based electrolytes.^[^ ^26^, ^27^, ^28^ ^]^ The presence of these ion pairs facilitates rapid ion migration to the anode surface, with SSIPs enhancing bulk ionic conductivity by maintaining higher ion dissociation and mobility, while CIPs promote interfacial charge transfer by reducing the desolvation barrier of lithium ions at the electrode. Simultaneously, the mesoporous architecture of SiO_x_ synergized with the conductive PCNF's uniform porous framework, which not only supplied abundant active sites for lithiation but also dramatically amplified reaction kinetics through synchronized ion‐electron migration. Our prior research revealed that Li^+^ insertion efficiency and kinetics are exquisitely governed by ion‐electron co‐conduction pathways.^[^ ^28^ ^]^ The synchronized transport of electrons and Li^+^ proves indispensable, as their initial concentrations dictate conduction trajectories and insertion velocities. Within the high‐concentration Li⁺ model, pre‐resided Li⁺ forged multiple parallel high‐speed electron highways, catapulting insertion rates exponentially. This mechanistic elegance perfectly explains Model 3′s rapid, uniform lithiation. Concurrently, PF_6_^‐^ and solvent decomposition yielded LiF, Li_2_CO_3_, and Li_2_O products that coalesced into an exceptionally stable and uniform inorganic‐rich SEI layer across fiber surfaces. This robust interfacial architecture markedly reduced anode impedance while suppressing electrolyte decomposition side reactions, ensuring flawless Li⁺ distribution. Empowered by these synergistic advantages, Model 3 achieved dramatically lower voltage requirements versus Model 2, attaining a remarkable ICE of 99.44% and reaching 0.81 V of open‐circuit voltage (OCV) at 5 V within merely 30 s (Figure 1f; Video S1, Supporting Information). Comparative analysis revealed that while both models utilized electric fields to accelerate Li⁺ migration, the lithium salt selection proved pivotal in determining Li⁺ desolvation dynamics, SEI layer composition/distribution, and ultimate lithiation efficiency. By employing LiPF_6_ prelithiation solution, Model 3 masterfully optimized Li⁺ migration behavior, orchestrating swift, uniform lithiation processes that eclipsed conventional performance benchmarks.

### 2.2 Materials Characterization Before and After Prelitiation in Different Models

**Figure 2:**
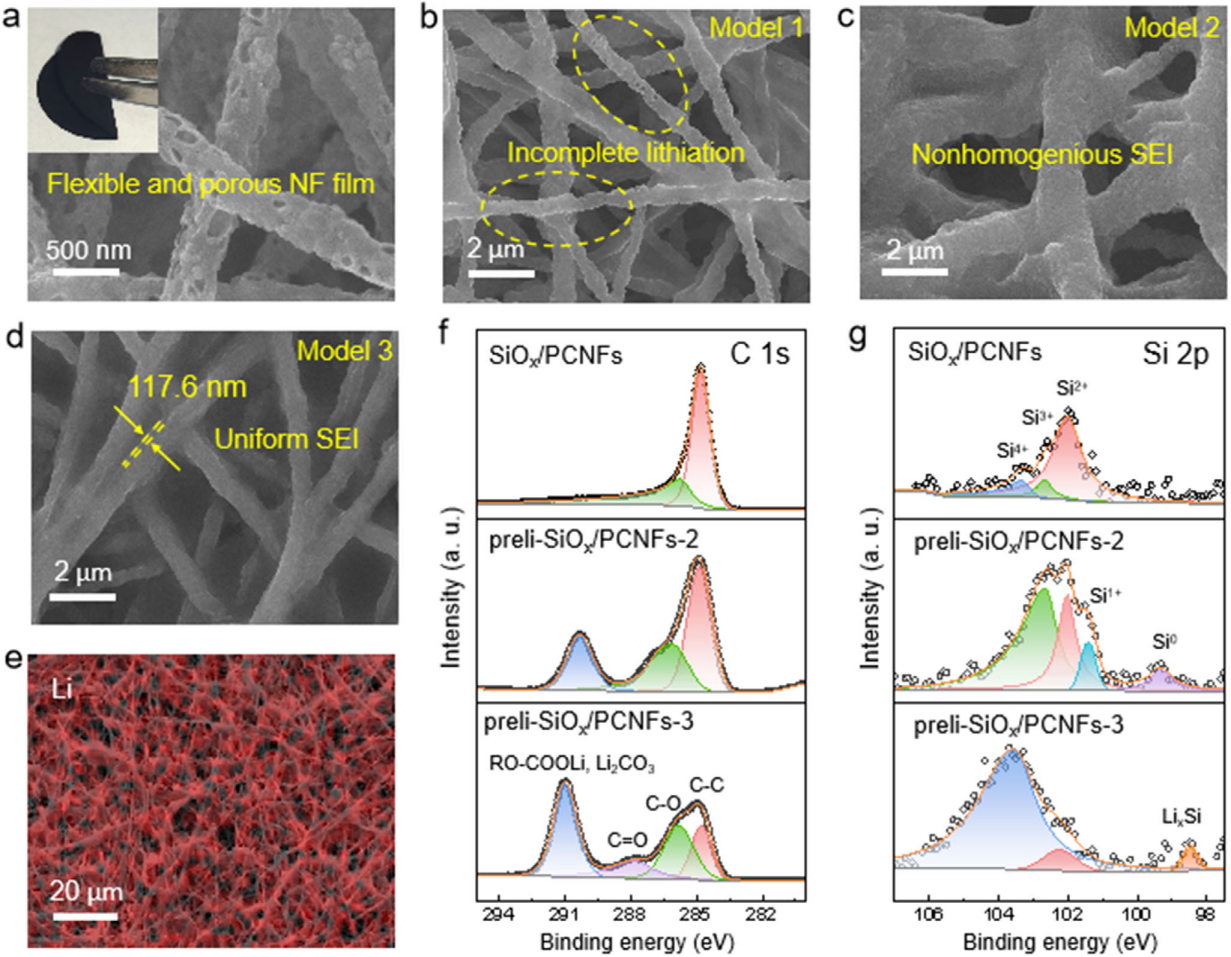
Materials characterization. SEM images of a) the initial SiO_x_/PCNF and b‐d) the prelithiated SiO_x_/PCNF using three different models. e) An AES image of Li distribution on the preli‐SiO_x_/PCNF‐3. f, g) High‐resolution C1s and Si 2p spectra of the SiO_x_/PCNF film, preli‐SiO_x_/PCNF‐2, and preli‐SiO_x_ /PCNF‐3.

Distinct lithiation pathways profoundly influence both the composition of the SEI and the morphological evolution of lithiated SiO_x_/PCNF anodes. Conventional coating methods yield carbon‐silicon anodes with densely packed structures, exhibiting low porosity and restricted SSA. This structural constraint impedes Li^+^ penetration and uniform intercalation, causing electrolyte decomposition to concentrate on the surface and fostering a thick, uneven, and mechanically unstable SEI layer (Figure S3a‐e, Supporting Information). Therefore, the ICE was much inferior (Figure S3f, Supporting Information). Addressing this challenge, we engineered flexible SiO_x_/PCNF films via electrospinning, featuring a meticulously uniform pore architecture (**Figure** **2**a). The resulting topological SiO_x_/PCNF film – embedded with 37.55 wt.% mesoporous SiO_x_ nanoparticles (NPs, average pore diameter: 2.9 nm) interlaced within a porous nanofiber network – achieved a remarkable SSA of 467.0517 m^2^ g^−1^ (Figure S4, Supporting Information). This hierarchically porous design drastically shortens Li^+^ diffusion pathways while supplying abundant electrochemically active sites, synergistically accelerating ion transport kinetics.^[^ ^29^ ^]^ Furthermore, the PCNF framework's optimized graphitization degree elevates electronic conductivity, diminishes charge‐transfer resistance, and guarantees synchronized Li^+^ and electron flux throughout cycling.

The morphological characteristics of prelithiated SiO_x_/PCNF under three distinct topological models were initially examined via scanning electron microscopy (SEM). In Model 1, insufficient lithiation was observed in preli‐SiO_x_/PCNF‐1 after a 30‐minute prelithiation period (Figure 2b), with suboptimal performance attributed to time‐intensive processing and unstable SEI formation (Figure S5, Supporting Information). Transitioning to Model 2, dramatic SEI infiltration into intra‐ and inter‐fiber pores occurred within merely 40 s. However, this approach produced an irregularly thickened SEI layer that roughened fiber surfaces (Figure 2c), likely due to excessive generation of irregular fluffy organic compounds, such as alkyl lithium carbonate, and an unstable SEI layer. Model 3 emerged as superior, establishing an intricately braided Li^+^/e^‐^ conductive network (Figure 2d) capped by a remarkably thin, 117.6 nm brown SEI film (Figure S6, Supporting Information). The energy‐dispersive X‐ray spectroscopy (EDS) and transmission electron microscopy (TEM) analyses of preli‐SiO_x_/PCNF‐3 revealed well‐preserved nanofiber pore architectures alongside a uniformly distributed SEI layer rich in C, P, F, and O elements (Figure S7, Supporting Information). This optimized structure enabled accelerated Li^+^ mobility through shortened diffusion pathways while maintaining electrode‐wide ionic homogeneity. Enhanced electrode‐electrolyte interfacial contact substantially diminished localized current density during prelithiation, curbing regional overpotential and parasitic reactions. Consequently, a compact, stable SEI layer with exceptional uniformity was achieved, as evidenced in Figure 2e and Figure S8 (Supporting Information).

Furthermore, the graphitized PCNF framework significantly enhanced electronic conductivity, bolstering the efficiency of electron supply at the electrode/electrolyte interface while diminishing potential differences. This synergistic effect substantially lowered the energy barrier for charge transfer. Simultaneously, the framework established a robust conductive network that seamlessly interconnected active NPs, effectively mitigating resistance arising from inadequate interparticle contact or interfacial imperfections. Consequently, this architecture enabled synchronized transport of Li^+^ and electrons, dramatically reducing charge transfer resistance and fostering homogeneous interfacial reactions. Atomic force microscopy (AFM) analyses conclusively demonstrated the exceptional smoothness of the lithiated NF film surface (Figure S9, Supporting Information), with surface roughness quantified through the contour arithmetic mean deviation (Ra) plunging to 187 nm – a remarkable improvement over both preli‐SiO_x_/PCNF‐2 and pristine SiO_x_/PCNF specimens. This dramatic reduction in Ra values directly reflected the optimized structural integrity of the SiO_x_‐based anode, successfully counteracting the destructive structural collapse typically induced by severe volume expansion and cyclic lithiation/delithiation stresses. The ingeniously engineered topological architecture facilitated the creation of a high‐performance interfacial conductive network, achieving dual objectives: alleviating mechanical stress concentrations to enhance structural durability while minimizing interfacial resistance.^[^ ^30^ ^]^ Collectively, these advancements propelled both the efficiency and operational stability of the prelithiation process to unprecedented levels.

To shed light on the distinct impacts of topochemical lithiation Models 2 and 3 on anode surface chemistry and bonding configurations, we conducted comparative XPS and Raman analyses of SiO_x_/PCNF, preli‐SiO_x_/PCNF‐2, and preli‐SiO_x_/PCNF‐3. The C 1s spectra (Figure 2f) revealed four characteristic peaks at 284.8, 285.8, 287.77, and 290.9 eV, corresponding to C‐C, C‐O, C = O, RO‐COOLi, and Li₂CO₃ species, respectively. Notably, preli‐SiO_x_/PCNF‐3 exhibited marked reduction in C‐C bonds alongside striking intensity enhancement in RO‐COOLi and Li₂CO₃ signatures, evidencing a more complete lithiation process and formation of inorganic‐rich SEI layers that bolster interfacial stability. Deconvolution of Si 2p spectra (Figure 2g) unambiguously resolved six chemical states: Li_x_Si (98.47 eV), Si⁰ (99.33 eV), Si¹⁺ (101.41 eV), Si^2^⁺ (102.01 eV), Si^3^⁺ (102.64 eV), and Si⁴⁺ (103.33 eV), directly contrasting the surface chemical landscapes shaped by the two lithiation protocols. Crucially, the Si^2^⁺, Si^3^⁺, and Si⁴⁺ states in pristine SiO_x_/PCNF aligned with SiO, SiO₁.₅, and SiO₂ configurations respectively, confirming the material's fundamental identity as a heterogeneous mixture of Si and SiO₂. This structural duality found validation in HR‐TEM imaging, where crystalline domains within SiO_x_/PCNF displayed 0.248 nm lattice fringes matching the (101) planes of cristobalite SiO₂ (Figure S10a, Supporting Information), while XRD patterns (Figure S10b, Supporting Information) concurrently revealed broad amorphous halos superimposed with weak crystalline reflections. The absence of detectable silicon phase in both XPS and XRD profiles likely stems from its sub‐detection‐threshold concentration. Collectively, these findings paint a portrait of SiO_x_/PCNF as a partially crystalline structure where ordered SiO₂ nanocrystals remain encapsulated within an amorphous host matrix.

Following 30 s of simultaneous lithiation, consistent with the SiO_x_ lithiation mechanism outlined in references,^[^ ^31^, ^32^ ^]^ Li^+^ and electrons notably targeted weaker Si‐O bonds in Model 2, preferentially generating low‐valent silicon. In striking contrast, Model 3 exhibited markedly advanced lithiation progression, as evidenced by distinct Li_x_Si phase signals in preli‐SiO_x_/PCNF‐3 alongside substantial Si⁴⁺ signatures from stabilized Li_x_SiO_y_ complexes, while conspicuously lacking intermediate silicon species responsible for excessive Li⁺ depletion. This observation was further corroborated by XRD mapping analysis (Figure S10c, Supporting Information), revealing lithium silicate derivatives (Li_2_Si_2_O_5_, Li_2_SiO_3_, and Li_4_SiO_4_) in preli‐SiO_x_/PCNF‐3 with characteristic peak broadening. The discernible low‐angle shift in SiO_2_ phase diffraction patterns indicated a notable expansion in lattice constants, conclusively demonstrating lithium‐induced crystalline restructuring through intercalation. These *in‐situ* formed lithium silicate networks not only significantly bolstered the anode's structural integrity and electrochemical resilience but also established a favorable foundation for subsequent SEI layer evolution during cycling. The Raman spectra comparison of the initial SiO_x_/PCNF and preli‐SiO_x_/PCNF‐3 also confirmed the crystalline structure changes caused by the prelithiation (Figure S11, Supporting Information).

The rapid and complete lithiation of Model 3 stemmed principally from LiPF₆’s superior ionic conductivity and moderately stable solvation shell compared to LiClO₄. The weaker interaction between PF₆⁻ anions and solvent molecules enabled swifter Li^+^ migration to the anode surface. While Li^+^ drove lithiation product formation, PF₆⁻ anions exhibited greater susceptibility to decomposition at the anode due to reduced pairing energies. Crucially, LiF derived from LiPF₆ decomposition (Figure S12, Supporting Information) formed a dense, stable inorganic SEI layer – this dual‐function barrier simultaneously suppressed electrolyte decomposition, enhanced electrochemical reaction kinetics,^[^ ^20^ ^]^ and mitigated volume expansion in SiO_x_/PCNF composites.^[^ ^33^ ^]^ Through these mechanisms, LiPF₆ in Model 3 achieved synergistic advantages: accelerated Li^+^ transport, exhaustive lithiation reactions, and efficient *in‐situ* SEI formation collectively minimized active Li⁺ loss during initial cycling, ultimately elevating ICE and fortifying overall electrochemical performance.

### 2.3 Electrochemical Characterization and SEI Analysis of Pre‐Lithiated Anodes

**Figure 3:**
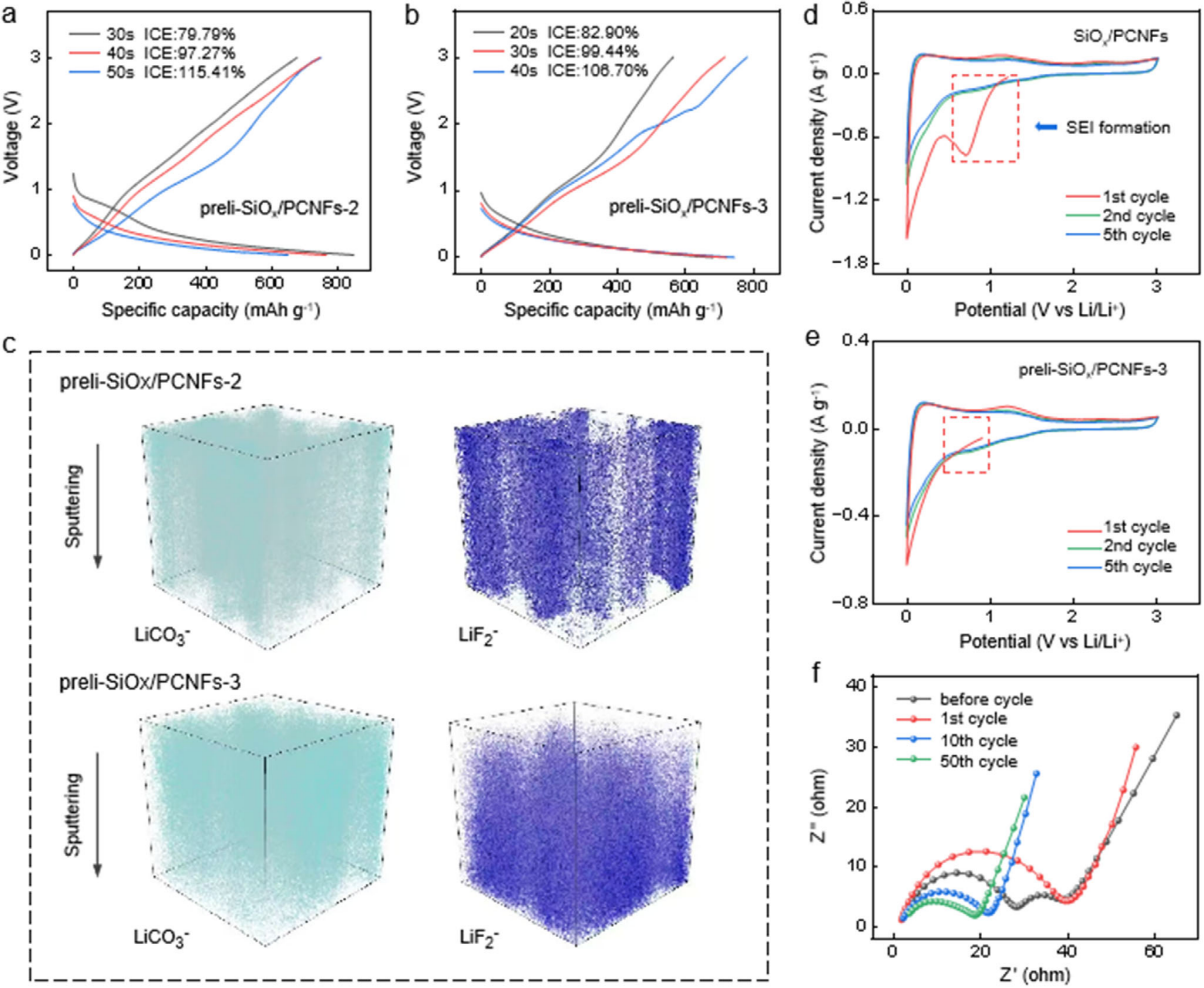
Electrochemical characterization and interface characteristic analysis of pre‐lithiated anodes. a,b) Initial cycle voltage profiles of preli‐SiO_x_/PCNF‐2 and preli‐SiO_x_/PCNF‐3 electrode. c) 3D structural views of ToF‐SIMS depth sputtering on the surface of preli‐SiO_x_/PCNF‐2 and preli‐SiO_x_/PCNF‐3. d,e) CV curves of the original and preli‐SiO_x_/PCNF‐3 anodes. f) Equivalent circuits of preli‐SiO_x_/PCNF‐3 half‐cells after 1, 10, and 50 cycles.

To optimize prelithiation models, initial charge‐discharge cycles of prelithiated anodes were systematically analyzed (**Figure** **3**a‐b; Figure S13, Supporting Information). Remarkably, the preli‐SiO_x_/PCNF‐2 anode demonstrated an ICE of 97.27% at 8 V, while the preli‐SiO_x_/PCNF‐3 anode achieved 99.44%, soaring to 106.70% when prelithiation time extended from 30 to 40 s. This superior performance stemmed from reduced Li^+^ migration resistance and exceptional lithiation kinetics in LiPF₆ solution. Notably, a pronounced plateau emerged in preli‐SiO_x_/PCNF‐2′s initial voltage curve (1.0‐0.5 V range post 30‐second lithiation), signaling incomplete SEI film formation—a vivid confirmation of Model 2′s suboptimal lithiation. While high ICE proves critical for Li‐ion batteries, excessively elevated values may amplify side reactions and lithium depletion in subsequent cycles, ultimately compromising longevity. Thus, the 30‐second lithiation window strikes an optimal equilibrium between efficiency and stability. These findings underscore the SEI layer's profound impact on battery kinetics and lithium utilization during prelithiation. To probe this relationship, the composition and evolution of SEI in Models 2 and 3 were meticulously examined through time‐of‐flight secondary ion mass spectrometry (ToF‐SIMS), enabling spatial mapping of lithium species in lithiated SiOx/PCNF anodes (Figure 3c; Figure S14, Supporting Information). Distinctive ion fragments – LiF₂⁻ (LiF marker), C₂H₂O⁻ (organic components), LiO⁻, and LiCO₃⁻ – revealed critical insights. Under LiPF₆‐regulated prelithiation, LiF₂⁻, LiCO₃⁻, and LiO⁻ exhibited intensified, homogeneous distribution, evidencing a denser and more uniform inorganic‐rich SEI layer. This structural coherence crucially decelerates Li^+^ and electrolyte consumption, directly correlating with enhanced battery durability—a conclusion further validated through parallel XPS investigations.

The nanoscale porous architecture, synergized with intrinsic nanoscale effects, played an instrumental role in governing these mechanisms. The ultrafine dimensions of the porous framework endowed the structure with an expansive SSA, dramatically enhancing interfacial contact between electrode and electrolyte. This augmented interface enabled meticulously uniform electrolyte decomposition during prelithiation. Furthermore, nanoconfinement effects amplified surface reactivity, accelerating ion diffusion kinetics while steering reaction pathways toward stable inorganic phase formation and disfavoring unstable organic byproducts. Kinetically, for alloying and conversion reactions, the temporal span required for reaction front propagation through NPs—culminating in complete phase transformation—proved substantially abbreviated in NPs versus bulk counterparts, powerfully underscoring the supremacy of nanoscale architectural control.^[^ ^34^ ^]^ The 3D continuous pore network guaranteed spatially homogeneous reaction distribution across the electrode‐electrolyte boundary, effectively mitigating localized parasitic reactions and optimizing SEI layer uniformity.^[^ ^20^ ^]^ Notably, while both systems exhibited organic constituents in SEI layers, preli‐SiO_x_/PCNF‐2 demonstrated markedly elevated organic content, corroborating the thesis that Model 2 developed a thicker SEI dominated by loosely structured organic matrices. Collectively, these insights illuminate nanoscale engineering as a cornerstone strategy for tailoring SEI characteristics and elevating lithium‐ion battery performance metrics.

Cyclic voltammetry (CV) measurements were systematically performed on SiO_x_/PCNF, preli‐SiOx/PCNF‐2, and preli‐SiO_x_/PCNF‐3 electrodes (Figure 3d‐e; Figure S15, Supporting Information) at a scan rate of 0.1 mV s^−1^ to elucidate reaction mechanisms during electrochemical processes. As shown in Figure 3d, a distinct irreversible peak appeared at ≈0.8 V during the initial discharge, indicating that the formation of the SEI and the decomposition of the electrolyte consumed a significant quantity of Li‐ions, resulting in high irreversible capacity. In Model 3 (Figure 3e), this reduction peak completely disappeared in subsequent cycles, and the peak shape became more stable, suggesting the formation of a complete and dense SEI layer. The redox peak at ≈0.1 V corresponded to the alloying/dealloying reaction between Si and Li‐ions, forming reversible Li_x_Si, which ensured the high capacity and ICE of the preli‐SiOx/PCNF‐3 anode.^[^ ^35^ ^]^ This reaction was crucial for silicon‐based anodes, as it allowed for the reversible storage of Li‐ions, which was a key factor in achieving high energy density and improved ICE in Li‐ion batteries. Furthermore, an anodic peak at ≈1.2 V was observed in all oxidation cycles, which might correspond to the reversible transformation of Li₂Si₂O₅ into SiO₂.^[^ ^36^ ^]^

To further confirm the ion intercalation kinetics, we measured the impedance and activation energy of the prelithiated anodes prepared in Model 2 and Model 3 (Figure S16a‐b, Supporting Information). Both the impedance and activation energy Ea of preli‐SiO_x_/PCNF‐3 were much smaller than that of preli‐SiO_x_/PCNF‐2. Through Galvanostatic Intermittent Titration Technique (GITT, Figure S16c‐d, Supporting Information) testing analysis, the diffusion coefficient (D_k_) value of preli‐SiO_x_/PCNF‐3 was higher than that of preli‐SiO_x_/PCNF‐2, indicating an enhanced Li^+^ transport kinetics in preli‐SiO_x_/PCNF‐3. These results confirm that different lithium salts do indeed affect the prelithiation effect. In addition, to prove the selection of salts and solvents is proper, we further explored the dynamic mechanism of Li^+^ diffusion behavior (Figure R17, Supporting Information), from which we obtained that the Li^+^ diffusion kinetics of preli‐SiO_x_/PCNF‐2 was mainly controlled by Li^+^ diffusion, while the Li^+^ diffusion kinetics of preli‐SiO_x_/PCNF‐3 was controlled by surface dominated pseudocapacitive behavior. The results further confirmed that the LiPF_6_ based prelithiation effectively reduces interfacial resistance, constructs a stable SEI film, and optimizes surface activity of the anode, significantly improving the kinetic performance.

Figure 3f presents the EIS of the preli‐SiO_x_/PCNF‐3 anode under different cycling conditions. According to the equivalent circuit fitting results shown in Figure S18 (Supporting Information), the impedance spectra include the electrolyte resistance (R_s_), SEI resistance (R_SEI_), and charge transfer resistance (R_ct_). In order to study the activation energy barrier and Li‐ion diffusion kinetics, we analyzed the evolution of R_ct_ and R_SEI_ during cycling. At the initial cycling stage, the R_SEI_ of preli‐SiO_x_/PCNF‐3 showed a significant reduction, suggesting that the initial formed dense inorganic SEI film effectively reduced the interfacial impedance and facilitated the Li‐ions transport and charge transfer. During cycling, the R_SEI_ stabilized, owing to the even distribution and structural robustness of the SEI film. This film effectively curbed the rise in Li‐ion diffusion resistance and alleviated interface damage stemming from the bulk effect. Compared with preli‐SiO_x_/PCNF‐2, the R_SEI_ of preli‐SiO_x_/PCNF‐3 was consistently lower and more stable (Table S1, Supporting Information). This was attributed to the doping of LiF and lithium silicate in its SEI, and these inorganic components enhanced the mechanical strength and chemical stability of the SEI, thus significantly improving the electrochemical performance of the anode. For preli‐SiO_x_/PCNF‐3, R_ct_ decreased from 11.27 Ω (first cycle) to 5.099 Ω (10th cycle) and stabilized at 6.39 Ω after 50 cycles, indicating enhanced transport kinetics of lithium ions. In contrast, preli‐SiO_x_/PCNF‐2 showed a sharp increase in R_ct_ from 3.717 Ω to 23.4 Ω over 50 cycles. This difference is attributed to the gradient lithium fluoride/lithium carbonate SEI layer (Figure 3c), where lithium fluoride provides a low‐energy pathway for Li^+^ migration (ionic conductivity ≈10^−8^ S cm^−1^), while the Li_x_SiO_y_ phase passivates surface defects and reduces the interfacial energy barrier. Although direct activation energy (𝐸_𝑎_) measurements were not performed, the suppressed R_ct_ growth is consistent with previous studies showing that lithium fluoride enriched SEI reduces 𝐸_𝑎_ by 30–50%. These results fully validate the advantages of Model 3 in electrochemical prelithiation and suggest that optimizing the composition and structure of SEI is key to enhance the anode performance.

### 2.4 Electrochemical Performance of Lithium‐Ion Batteries with the Prelithiated Anodes

**Figure 4:**
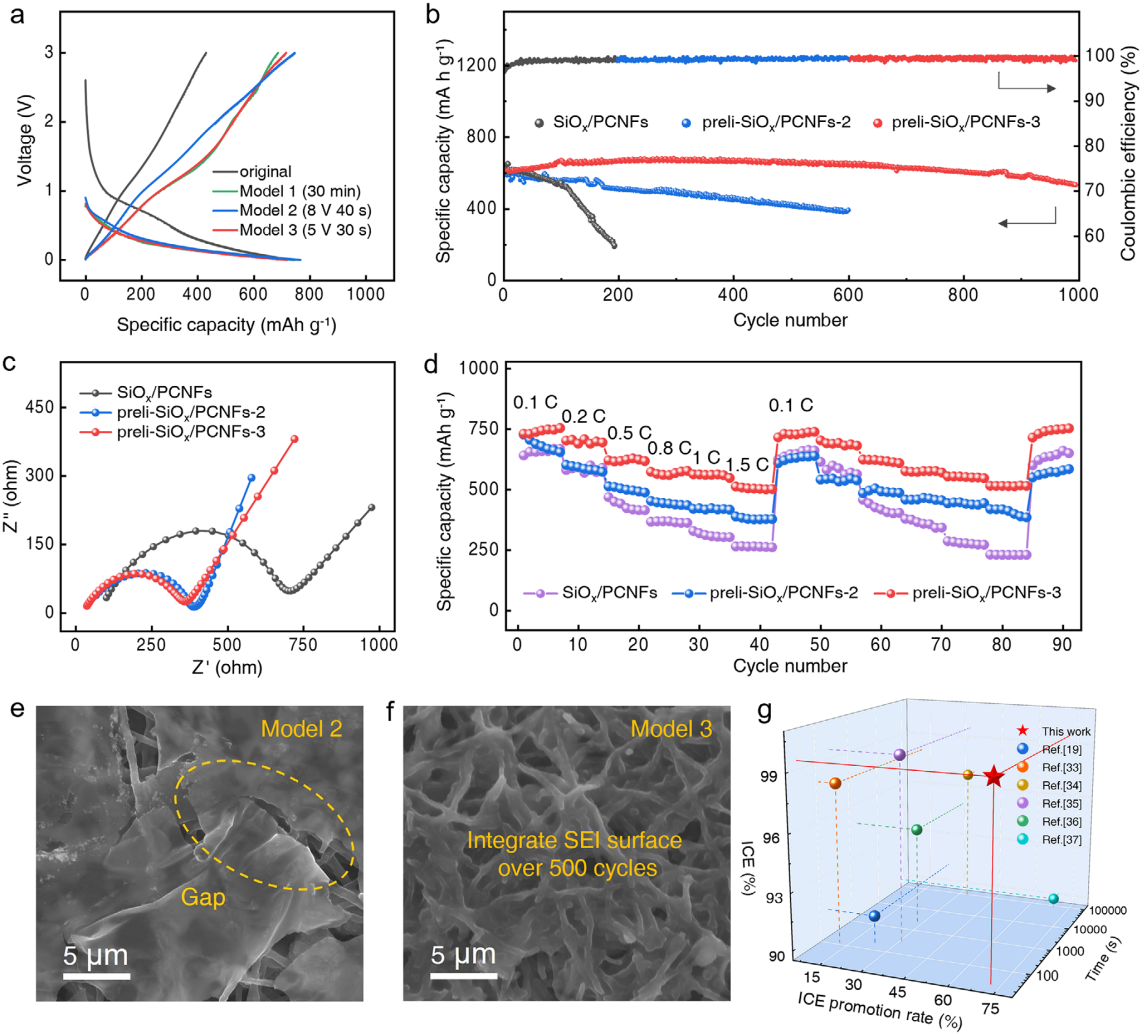
Electrochemical performance of Li‐ion batteries with the prelithiated anodes. a) Voltage profiles of SiO_x_/PCNF||Li and preli‐SiO_x_/PCNF||Li cells. b) Long cycling performance of SiO_x_/PCNF||Li and preli‐SiO_x_/PCNF||Li at 0.2 C. c) EIS tests of batteries after 1000 cycles with different anodes. d) Rate capability of SiO_x_/PCNF and preli‐SiO_x_/PCNF anodes. e, f) SEM images of preli‐SiO_x_/PCNF‐2 and preli‐SiO_x_/PCNF‐3 anodes after 500 cycles. g) Performance comparison of pre‐lithiation strategy between published literature and this work.

The voltage profiles in **Figure** **4**a vividly demonstrate the efficacy of prelithiation for preli‐SiOx/PCNF. All three prelithiation models remarkably boosted the ICE to above 90%, with Model 3 (5 V, 30 s) emerging as the most promising candidate due to its low voltage demand and ultrashort processing duration. This masterfully calibrated strategy seamlessly offset the irreversible capacity loss (285.193 mA h g⁻¹) induced by SEI formation. To probe the long‐term robustness of this technique, cycling stability tests were conducted on both prelithiated and non‐prelithiated SiO_x_/PCNF anodes in half‐cell configurations at 0.2 C (0–3 V), excluding initial activation cycles. As Figure 4b reveals, the preli‐SiO_x_/PCNF‐3||Li cell showcased exceptional capacity retention, preserving 78% of its capacity through 1000 cycles while maintaining Coulombic efficiency >99%—a stark contrast to the non‐prelithiated counterpart, which suffered rapid capacity fade and escalating interfacial resistance. Notably, the Model 1‐treated anode exhibited erratic capacity fluctuations post‐400 cycles, a deterioration rooted in structural collapse from incomplete prelithiation and perpetual SEI layer fragmentation‐regeneration cycles (Figure S19, Supporting Information).

Post‐1000 cycles, preli‐SiO_x_/PCNF‐3 proudly claimed the lowest RSEI and Rs values among all samples – a triumph orchestrated by its uninterrupted graphitized carbon matrix and inorganic‐dominant, uniform SEI layer (Figure 4c; Table S2, Supporting Information). The minimized Rs stemmed from the CNF architecture's precision‐engineered electron highways and optimized electrode‐electrolyte interfaces, while the suppressed R_SEI_ reflected the barrier‐like efficiency of the ultrathin, inorganic‐rich SEI in facilitating Li‐ion shuttling. Beyond cycling prowess, preli‐SiO_x_/PCNF‐3 demonstrated a dramatically enhanced rate capability, delivering 512.8 mA h g⁻¹ at 1.5 C before rebounding to 730.4 mA h g⁻¹ upon returning to 0.1 C (Figure 4d; Figure S20, Supporting Information). The nanoscale effect of mesoporous SiO_x_ NPs significantly shortened the Li^+^ diffusion path, while the PCNF framework provided efficient electron transport channels and formed reliable interfacial contact with SiO_x_ NPs, thereby achieving a synergistic effect between ion and electron transport. These structural and interfacial improvements collectively explain the remarkable cycling stability and superior rate performance of the preli‐SiO_x_/PCNF‐3 battery.

Figure S21 (Supporting Information) shows the SEM images of preli‐SiO_x_/PCNF‐2 and preli‐SiO_x_/PCNF‐3 after the first cycle, which further revealed the structural differences during the initial charge and discharge stage. Preli‐SiO_x_/PCNF‐2 showed obvious particle accumulation on the fiber surface after 1 turn of cycling (Figure S21a, Supporting Information), indicating that the formation of the SEI film was not uniform and the SEI film was thicker in some areas leading to the obstruction of Li‐ion transport. In contrast, preli‐SiO_x_/PCNF‐3 exhibited a more uniform surface coverage under the same condition, with the fiber structure remaining intact and a thinner and denser and smoother SEI film (Figure S21b, Supporting Information), demonstrating superior structural properties. With the operation of the battery, this high‐quality SEI film formed during the initial stage laid the foundation for long‐term cycling performance. After 500 cycles, the preli‐SiO_x_/PCNF‐2 anode had obvious cracks (Figure 4e), while the preli‐SiO_x_/PCNF‐3 retained its intact and self‐supported 3D conductive network structure (Figure 4f). The prelithiation significantly mitigated the volume expansion of SiO_x_ during lithium absorption by virtue of the inorganic nature of SEI and the nanopore structure inside the anode film, effectively maintaining the integrity of SEI and enhancing the cycling performance. In contrast, the unlithiated SiO_x_/PCNF lost its fibrous structure after 500 cycles, attributing to the spatial variation of the SEI leading to a greater concentration of current in local region. This induced reaction heterogeneity in the SEI growth, resulting in the generation of inhomogeneous SEI particles (Figure S22, Supporting Information). This behavior was characterized by significant SEI fracture and regeneration, unlike previous results.

Finally, by the performance comparison of prelithiation with the published literature (Figure 4g; Table S3, Supporting Information), it can be noticed that the proposed prelithiation strategy exhibited significant advantages in terms of the improvement of ICE and the reduction of processing time.^[^ ^19^, ^37^, ^38^, ^39^, ^40^, ^41^ ^]^ This simple and low‐cost lithium replenishment method not only effectively improved the ICE of the cell, but also significantly improved the lithiation efficiency and cycling stability, and its performance enhancement was better than most of the reported techniques.

## 3 Discussion

The carbon silicon anode faces significant Li^+^ depletion during initial cycling, severely compromising its ICE. Our research focused on a unique topological intercalation prelithiation strategy and fibrous topological anode architecture. Among three engineered prelithiation models, the ultrafast electrically‐driven Model 3 achieved great results: a 99.44% ICE within 30 s and remarkable 1000‐cycle stability – outperforming conventional sluggish prelithiation techniques. This result stems from topological fiber material engineering and precision‐controlled topochemical lithium infusion. We believe that this prelithiation method is more suitable for electrode materials with fiber structures, which may have more advantages in the application of flexible batteries. Because for flexible electrode materials, improving the energy density of batteries is more crucial. The ultrafast kinetics in the pre‐lithiation process can be attributed to several key factors. First, the ion‐pair distribution in the LiPF_6_ solution provides a high concentration of active Li^+^, which significantly lowers the energy barriers for Li^+^ diffusion. Second, the abundant pores and oxygen vacancies in the SiO_x_/PCNF serve as low‐energy‐barrier reaction sites, preferentially adsorbing and embedding Li⁺ to form a stable Li_x_SiO_y_ phase, which further reduces the activation energy of the interfacial reaction. Third, the porous structure of the PCNF enhances the permeability of the electrolyte and shortens the Li⁺ diffusion pathway. Together, these factors enable the pre‐lithiation process to achieve efficient ion insertion and interfacial stabilization in a short period of time.

**Figure 5:**
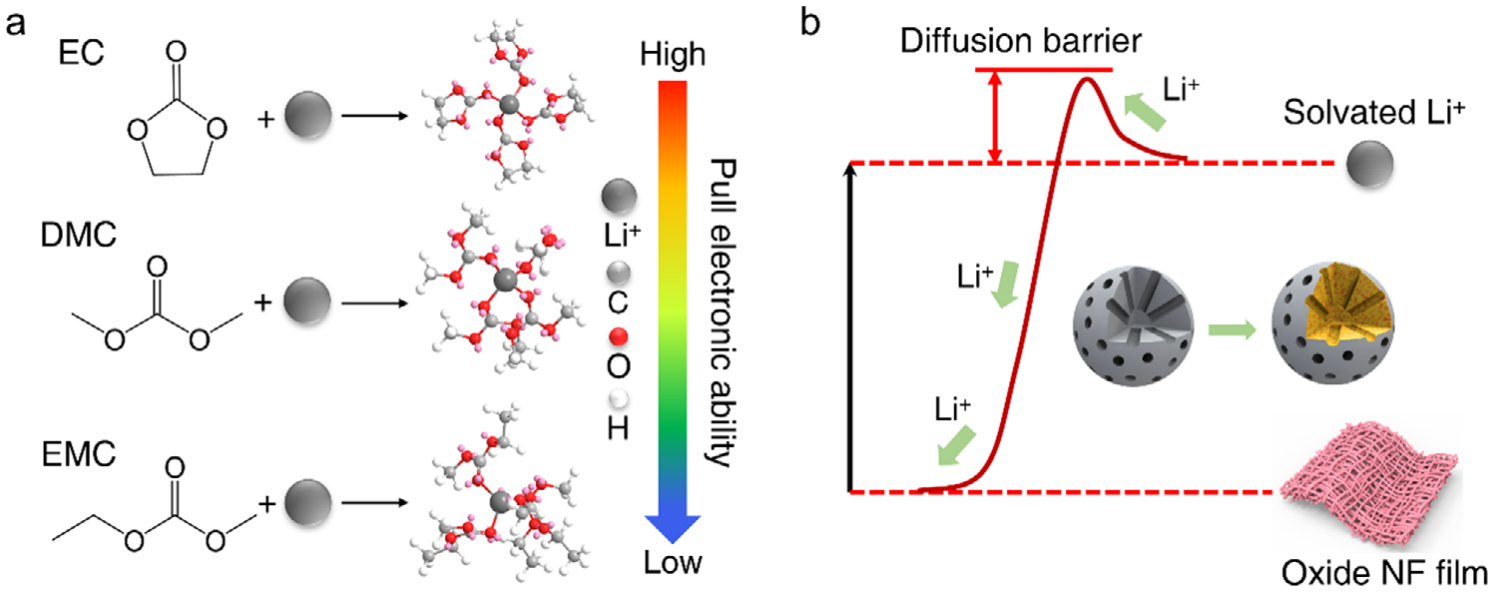
a) The electron withdrawing ability of solvents directly affects the intercalation reaction kinetics of dissolved lithium salts. b) Schematic diagram of electron movement between Li and SiO_2_.

Our investigation further deciphers the lithium‐intercalation orchestration behind SEI evolution. Comparative analysis of LiPF_6_ and LiClO_4_ electrolytes unveiled stark kinetic divergences: LiPF_6_’s CIP/SSIP‐dominant ion pairing and low coordination energy enable rapid lithium migration and preferential anion decomposition. This cascade yields a LiF‐rich, crystalline SEI matrix – a lithium superhighway that minimizes interfacial resistance. Conversely, LiClO_4_’s sluggish dissociation kinetics and high pairing energy generate porous, unstable SEI layers plagued by elevated impedance. In addition, our findings establish solvent selection as a master key for the fast prelithiation (**Figure** **5**a). Cyclic carbonate (EC), due to its high dielectric constant and strong molecular polarity, can form strong coordination interactions with Li^+^. In contrast, linear carbonates (such as DMC and EMC) have weaker polarity and consequently exhibit weaker interactions with Li^+^. Therefore, in lithium battery electrolytes, the solvation of Li^+^ is predominantly governed by EC. Under ambient conditions, when lithium metal contacts SiO_2_ directly, the high chemical stability and robust crystal lattice of SiO_2_ preclude the spontaneous diffusion of Li^+^ into its interior (Figure 5b). In other words, no spontaneous insertion or conversion reactions occur, and an external voltage is required to drive these processes. The precise voltage depends on factors such as the phase of SiO_2_ (crystalline versus amorphous), the presence of surface defects, and interfacial conditions. Therefore, to effectively facilitate the diffusion or insertion of Li^+^ into SiO_2_ via electrochemical methods, a voltage must be applied to overcome these barriers.

## 4 Conclusion

In conclusion, this study solved the key problem of SiO_x_/C anode through a topological intercalation prelithiation strategy. The flexible SiO_x_/PCNF anode, engineered with a nanoscale mesoporous architecture and meticulously uniform pore distribution, achieved remarkably rapid Li^+^ transport, effectively mitigated volume expansion, and preserved structural integrity throughout cycling. By unraveling the interplay between LiPF_6_/LiClO_4_ decomposition kinetics and SEI formation dynamics, we demonstrated that LiPF_6_ facilitated the accelerated formation of a dense inorganic SEI layer, leveraging its lower pairing energies and unique ion‐pairing behavior dominated by CIP and SSIP configurations. These synergistically amplified structural and functional advantages enabled an ultrafast, electrically‐driven prelithiation process—achieving in‐situ construction of a robust 3D Li‐ion enriched electron‐conducting network within merely 30 s. This breakthrough yielded an unprecedented ICE of 99.44% while demonstrating exceptional cycling stability exceeding 1000 cycles. These insights establish groundbreaking theoretical frameworks for carbon‐silicon composite anode design and illuminate scalable pathways for advanced prelithiation methodologies.

## 5 Experimental Section

### Electrode Preparation

The preparation of mesoporous SiO_x_ NPs commenced with the following procedure: 480 mL of deionized water (DIW), 3.5 mL of a 2 M Sodium Hydroxide (NaOH) solution, 0.6 g of cetyltrimethyl ammonium bromide (CTAB), and 5 mL of Tetraethylorthosilicate (TEOS) were stirred vigorously at 40 °C for 2 h. The resulting SiO_x_ NPs were collected via vacuum filtration and subsequently calcined at 700 °C for 3 h in an air atmosphere to remove the CTAB template. Finally, the calcined NPs were ball‐milled for 1 h to obtain uniformly dispersed mesoporous SiO_x_ NPs. The preparation of SiO_x_/PCNF films via electrospinning and annealing processes was conducted as follows: An appropriate quantity of SiO_x_ NPs was uniformly dispersed in DIW using a powerful sonication bath for 2 h. Subsequently, polyvinyl alcohol (PVA) powder (10 wt.%) and polytetrafluoroethylene (PTFE, 60% dispersion) were introduced into the suspension and stirred for 8 h. The electrospinning process was executed with an applied voltage of 20 kV and a distance of 20 cm between the needle and the rotating collector. The temperature and humidity within the electrospinning chamber were maintained at 25 ± 5 °C and 50 ± 5%, respectively. The initially spun PVA NFs were dried under vacuum at 70 °C for 2 h to ensure complete removal of the remaining DIW. The membrane was subsequently heat‐treated at 220 °C for 2 h to pre‐oxidize the PVA. Finally, the membrane was annealed at 1000 °C for 2 h in a high‐purity N_2_ atmosphere (99.999%).

### Electrochemical Prelithiation

Three pre‐lithiation models were designed to validate the proposed strategy. Specifically, SiO_x_/PCNF membranes and circular Li sheets were clamped and placed on both sides of an electrolytic cell containing different solvents. Models 1 and 3 utilized a 1 M LiPF_6_ solution, while model 2 employed a 1 M LiClO_4_ solution, both in a mixed solvent of EC: DMC: EMC with a volume ratio of 1:1:1. Model 1 involved a wire to directly connect the SiO_x_/PCNF membrane and the Li sheet, whereas models 2 and 3 used voltage output devices to connect the SiO_x_/PCNF membrane and Li sheet. The lithiated SiO_x_/PCNF was rinsed with DMC to eliminate any residual electrolyte.

### Electrochemical Characterizations

The electrochemical performance of the anodes was examined using a coin‐type half‐cell (type 2025 R). The cell assembly was completed in an Ar‐filled glove box (with H_2_O and O_2_ levels below 0.01 ppm) using Li‐metal as the counter electrode. The electrolyte salt consisted of 1 M LiPF_6_, and the electrolyte solvent was a mixture of EC/DMC/DEC of 1:1:1 v/v with 10 vol% fluorinated ethylene carbonate (FEC) added. The assembled battery was left to stand for 12 h to ensure full wetting of the electrolyte and to reduce polarization. CV curves were obtained from a half‐cell, with five cycles scanned between 0 and 3.0 V at a rate of 0.1 mV∙s^−1^ (versus Li^+^/Li). Cycling and rate performance were measured by the Neware Battery Test System at 25 °C (0‐3.0 V). EIS was collected at 60% depth of discharge (DOD) on a Zahner Elektrik IM6 electrochemical workstation over the frequency range of 10 mHz to 100 kHz, using an alternating voltage of 5 mV. All tests were conducted at room temperature (25 °C).

### Physical Characterizations

The structures of materials were characterized by field emission SEM (Hitachi S‐4800) and HRTEM (JEM‐2100F). The crystal and chemical structures were checked via Bruker XRD with Cu Kα radiation between 5° and 90°, XPS (PHI 5000C ESCA System), and Raman (LabRAM HR Evolution). A BET analyzer (ASAP 2460, Micromeritics Co., U.S.A.) was conducted to calculate the SSA of samples. ToF‐SIMS (ToF‐SIMS 5, ION TOF) was used to analyze the surface chemical structure and for depth profiling. The AFM system (Bruker Corp., Dimension Icon) was used to measure the surface morphology of the original sample and the lithiated SEI.

## Conflict of Interest

The authors declare no conflict of interest.

## Author Contributions

All authors contributed to discussing and revising the paper.

## Supporting information



Supporting Information

Supporting Information

## Data Availability

The data that support the findings of this study are available from the corresponding author upon reasonable request.
