# Supplementary material for: An Ultrafast Charge‐Driven Topological Intercalation Prelithiation Strategy for Carbon‐Silicon Composite Anodes
Source: Adv Sci (Weinh). 2025 Jun 4;12(32):e06636. doi: 10.1002/advs.202506636 (PMC12407273; doi:10.1002/advs.202506636)
Supplement: Supplementary file 1 — Supporting Information [file ADVS-12-e06636-s002.pdf]

ADVANCED SCIENCE
Open Access

Supporting Information

for *Adv. Sci.*, DOI 10.1002/advs.202506636

An Ultrafast Charge-Driven Topological Intercalation Prelithiation Strategy for Carbon-Silicon Composite Anodes

Yifan Zhao, Liang Zhang, Qian Liu, Mingyu Liu, Jiajia Shen, Hui Ma, Juejing Dai, Xi Yu and Jianhua Yan**

Supporting Information

An Ultrafast Charge-Driven Topological Intercalation Prelithiation Strategy for Carbon-Silicon Composite Anodes

Yifan Zhao[1], Liang Zhang[1], Qian Liu[1], Mingyu Liu[3], Jiajia Shen[2], Hui Ma[2], Juejing Dai[3], Xi Yu[3*], Jianhua Yan[1,2,3*]

[1]*College of Textiles, Donghua University, Shanghai 201620, China.*

[2]*College of Material and Textile Engineering, Jiaxing University, Zhejiang, 314001, China*

[3]*School of Textile Materials and Engineering, Wuyi University, Jiangmen, 529020, China*

***Corresponding Authors**: Prof. Jianhua Yan (yanjianhua@dhu.edu.cn)

Prof. Xi Yu (j002752@wyu.edu.cn)

Supporting Figures

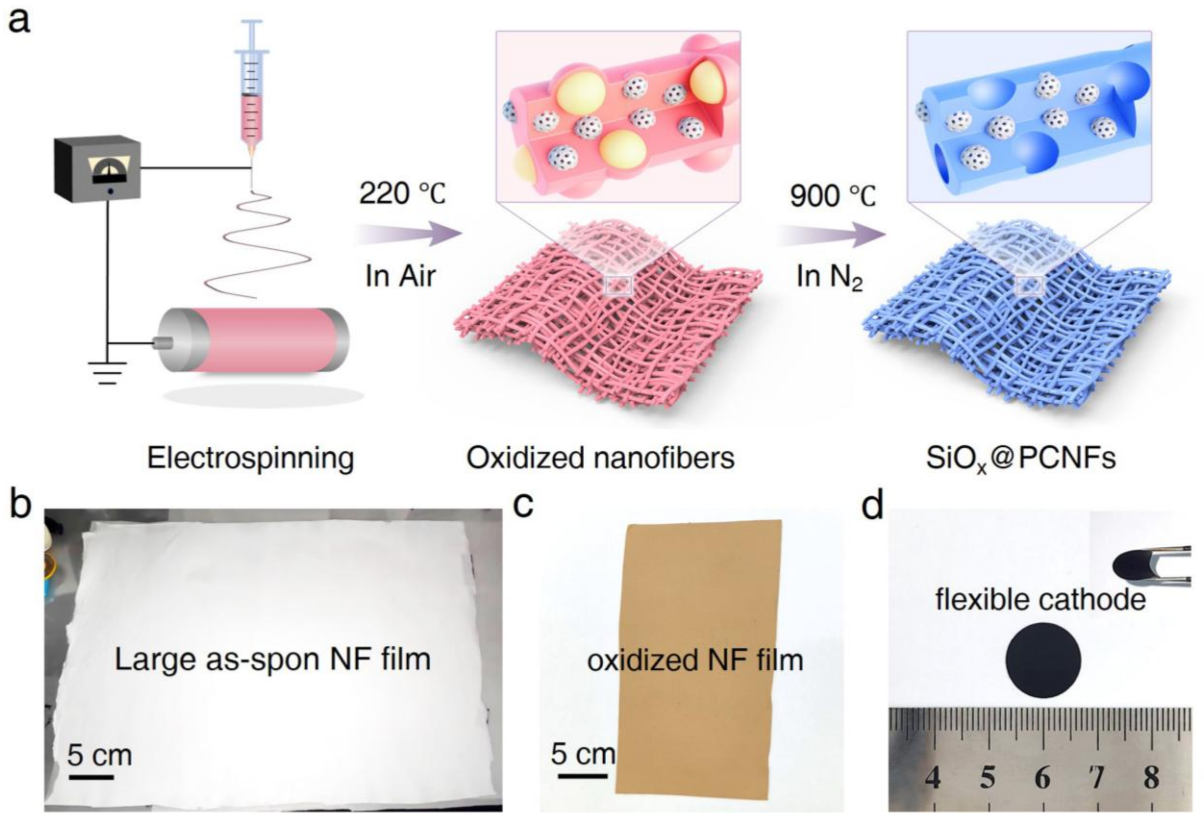

Figure S1. The preparation of flexible SiO_x/PCNF films. (**a**) The schematic diagram of flexible SiO_x/PCNF thin films prepared by electrospinning and annealing in our laboratory. The porosity and fiber diameter could be fine-tuned by adjusting the applied voltage, the viscosity and conductivity of the sol, and the distance between the needle and the receiving plate. (**b**) A large flexible SiO_x/PTFE@PVA electrospun original film manufactured by a trial production machine. (**c**) Flexible SiO_x/PTFE@PVA fiber membrane after oxidation. (**d**) The flexible conductive black SiO_x/PCNF thin film anode was prepared by an annealing technique.

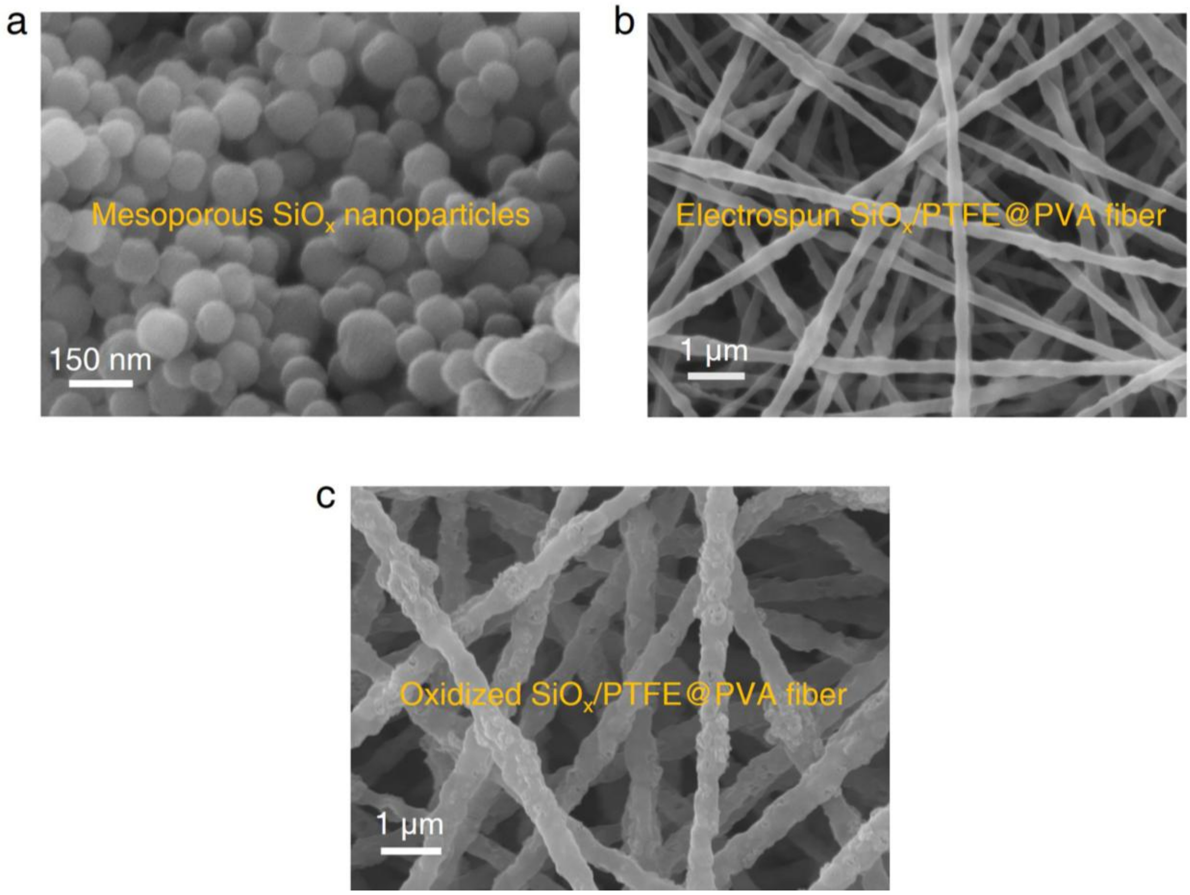

Figure S2. The SEM images of mesoporous SiO_x NPs and flexible fibre films. (**a**) The SEM image of mesoporous SiO_x NPs. (**b**) SEM image of electrospun SiO_x/PTFE@PVA fiber membrane. (**c**) SEM image of oxidized flexible SiO_x/PTFE@PVA fiber membrane.

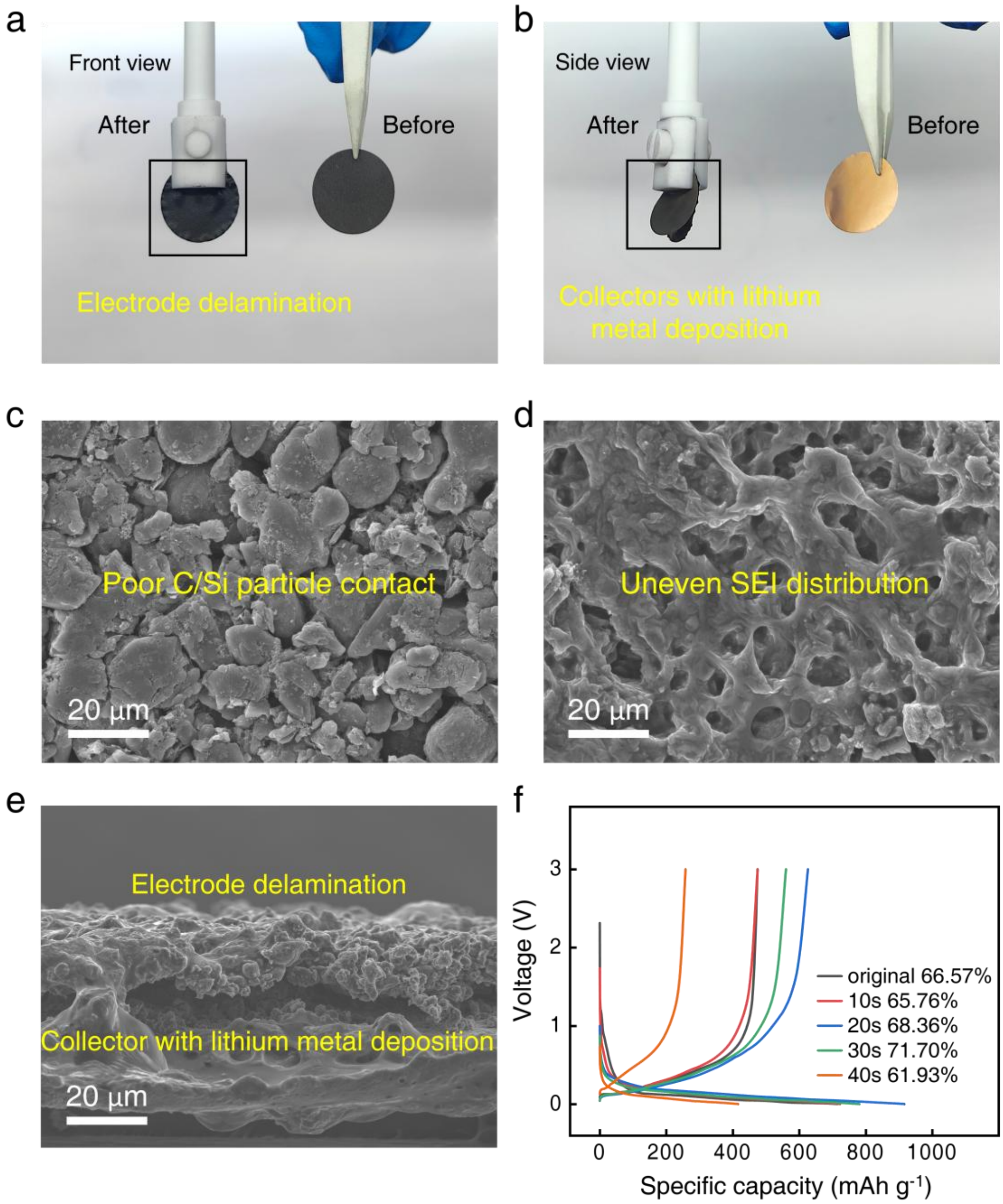

Figure S3. Characterization of the Prelithiation of commercial C/Si anodes. (**a-b**) Demonstration of commercial C/Si anodes before and after pre-lithiation. (**c**) SEM image of the pristine commercial C/Si anodes. (**d-e**) Front-view and side-view SEM images of the commercial C/Si anodes after electrochemical prelithiation (5 V, 30 s). (**f**) Initial voltage profile and ICE of prelithiated commercial C-Si electrodes in half-cells.

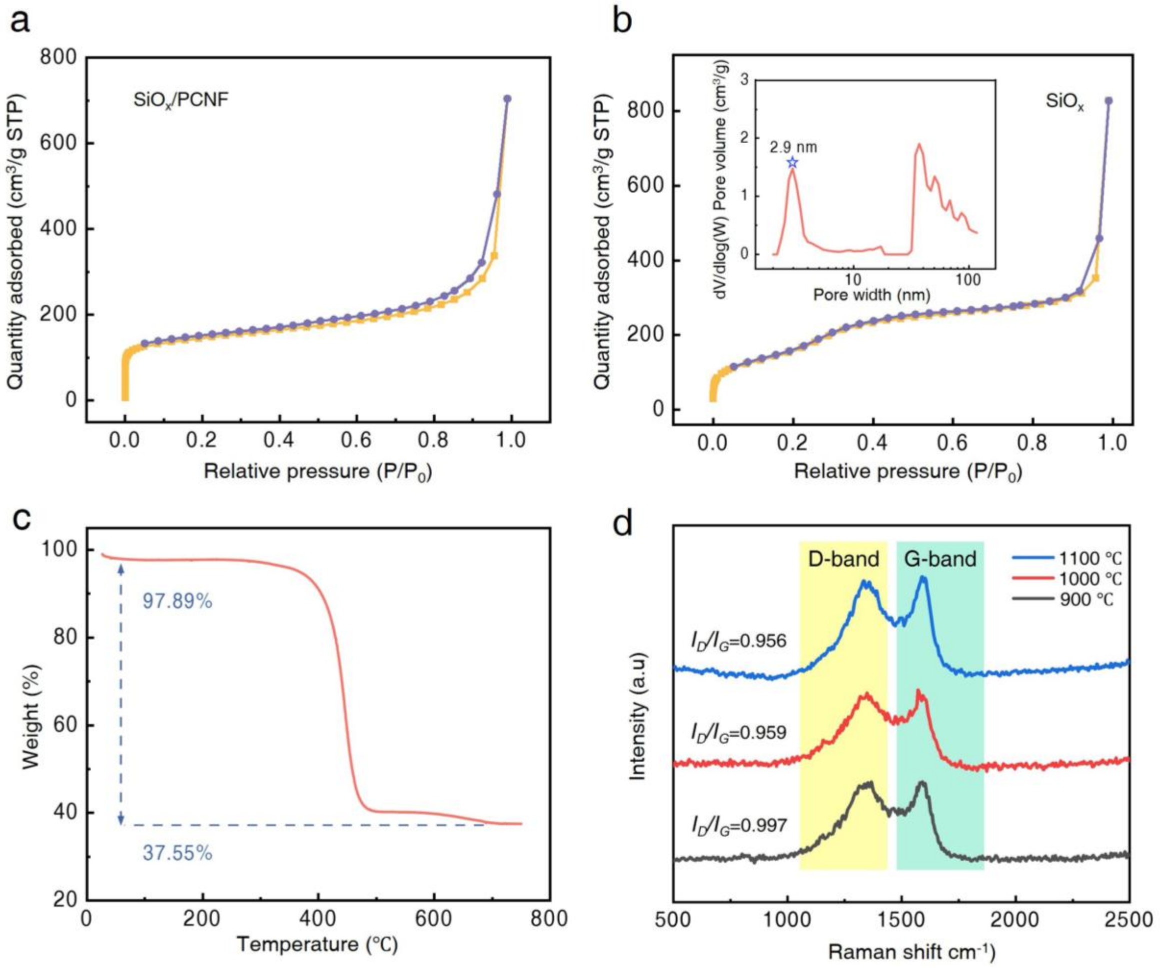

Figure S4. Structural and compositional analysis of flexible SiO_x/PCNF films. (**a**) N_2-adsorption/desorption isotherms of SiO_x/PCNF films. (**b**) N_2-adsorption/desorption isotherms and pore size distribution of mesoporous SiO_x NPs. (**c**) TG curve of SiO_x/PCNF in air. The decomposition of carbon fiber skeleton above 400 °C showed that the SiO_x content in SiO_x/PCNF was 37.55 wt.%. (**d**) Raman spectra of SiO_x/PCNF samples with different annealing temperatures. The 1000 °C annealing condition was the optimal taking into account the degree of graphitization and flexibility of the sample.

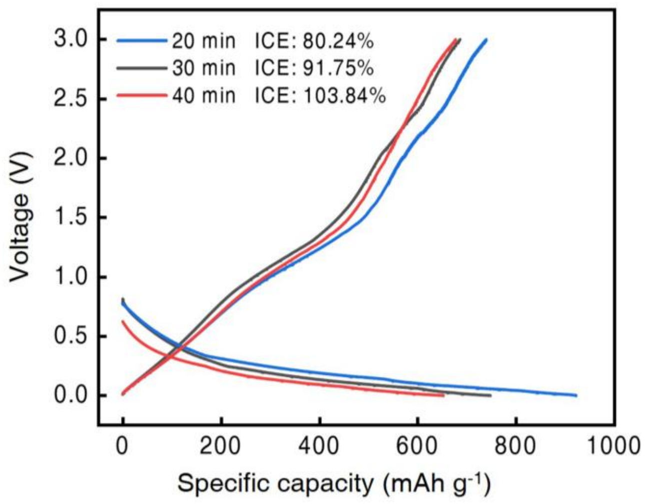

Figure S5. Voltage profiles of preli-SiO_x/PCNF with different lithiation times using Model 1 strategy.

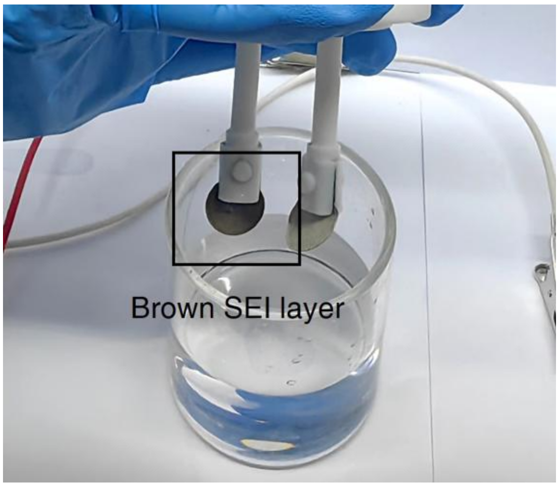

Figure S6. Digital photo of the preli-SiO_x/PCNF-3 with brown SEI layer.

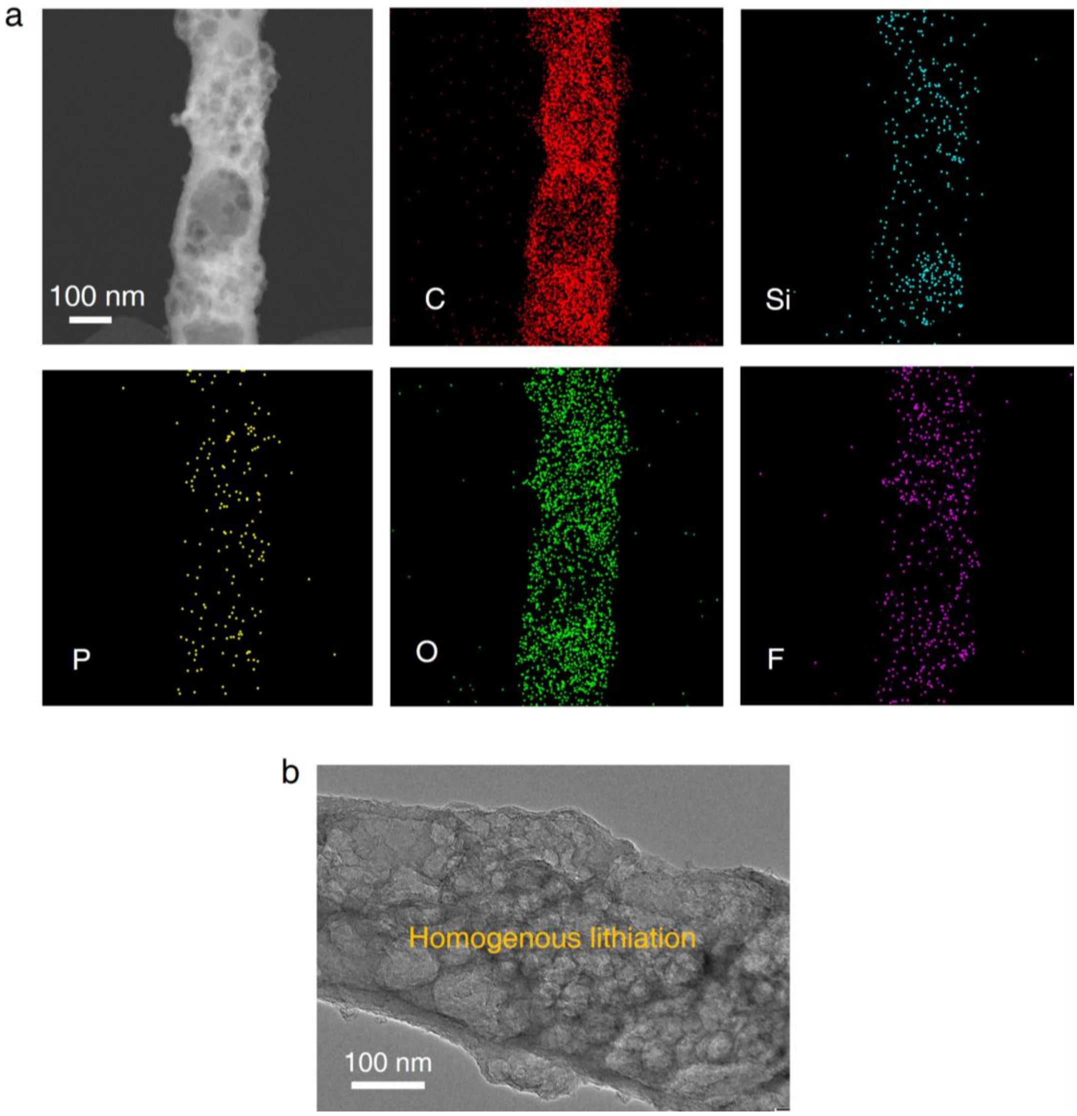

Figure S7. EDS and TEM characterizations of preli-SiO$_x$/PCNF-3. (**a**) EDS mapping images of C, Si, P, O, F elements and (**b**) TEM image for lithium anode preli-SiO$_x$/PCNF-3.

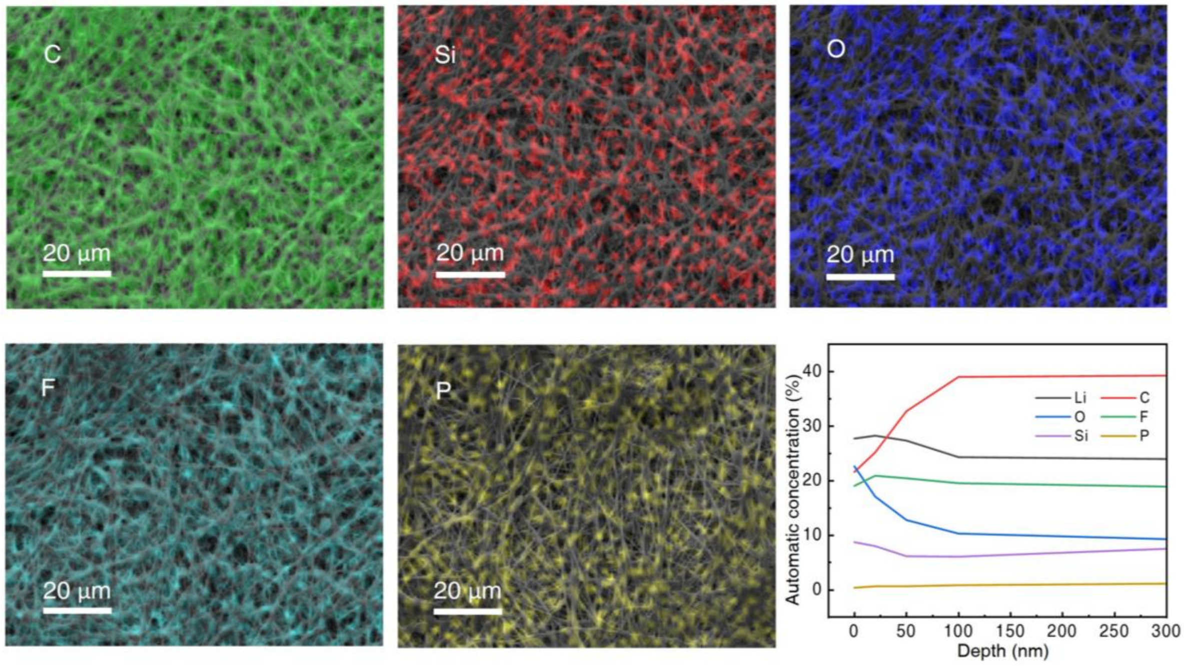

Figure S8. AES mapping image and depth analysis map of C, Si, O, F, P elements for lithium anode preli-SiO$_x$/PCNF-3. As the etching depth increases, the Li elemental content firstly decreases and remains stable after 100 nm, indicating that the fiber film is also uniformly lithiated in the depth direction.

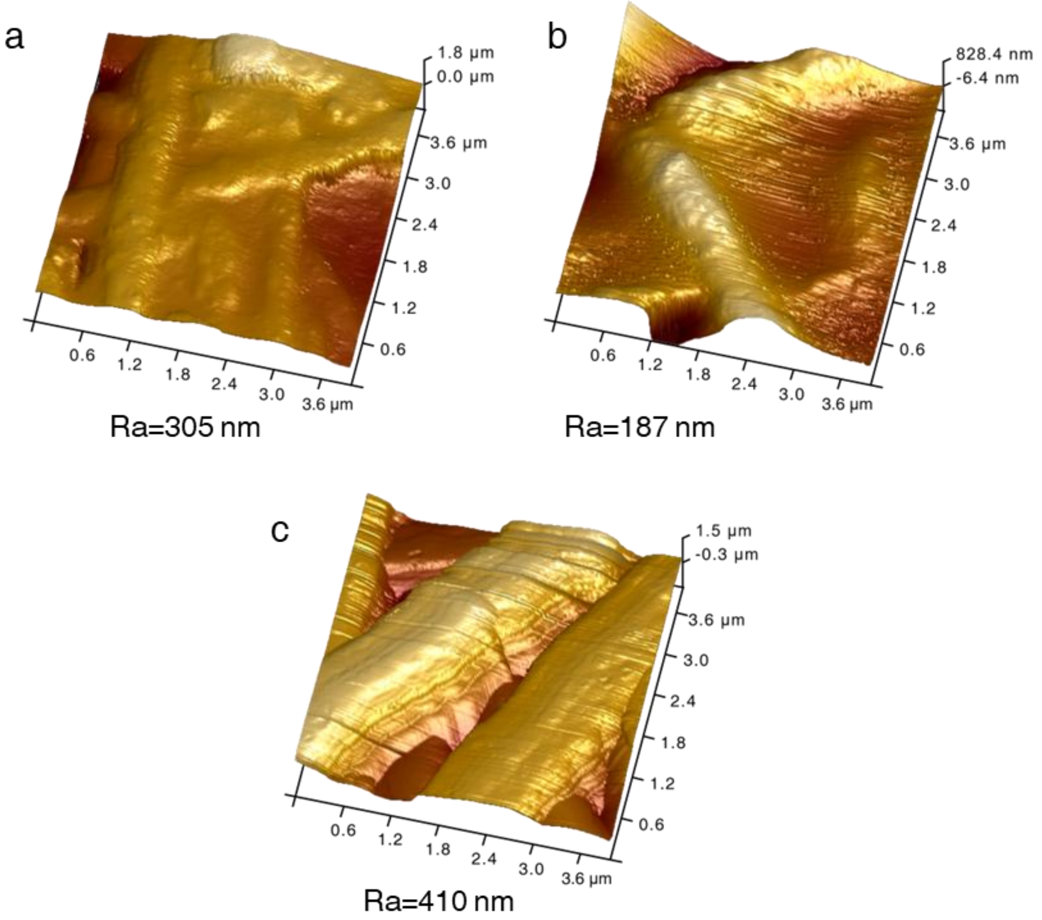

Figure S9. 3D AFM images of (**a**) preli-SiO_x/PCNF-2, (**b**) preli-SiO_x/PCNF-3 and (**c**) fiber membrane without pre-lithiation.

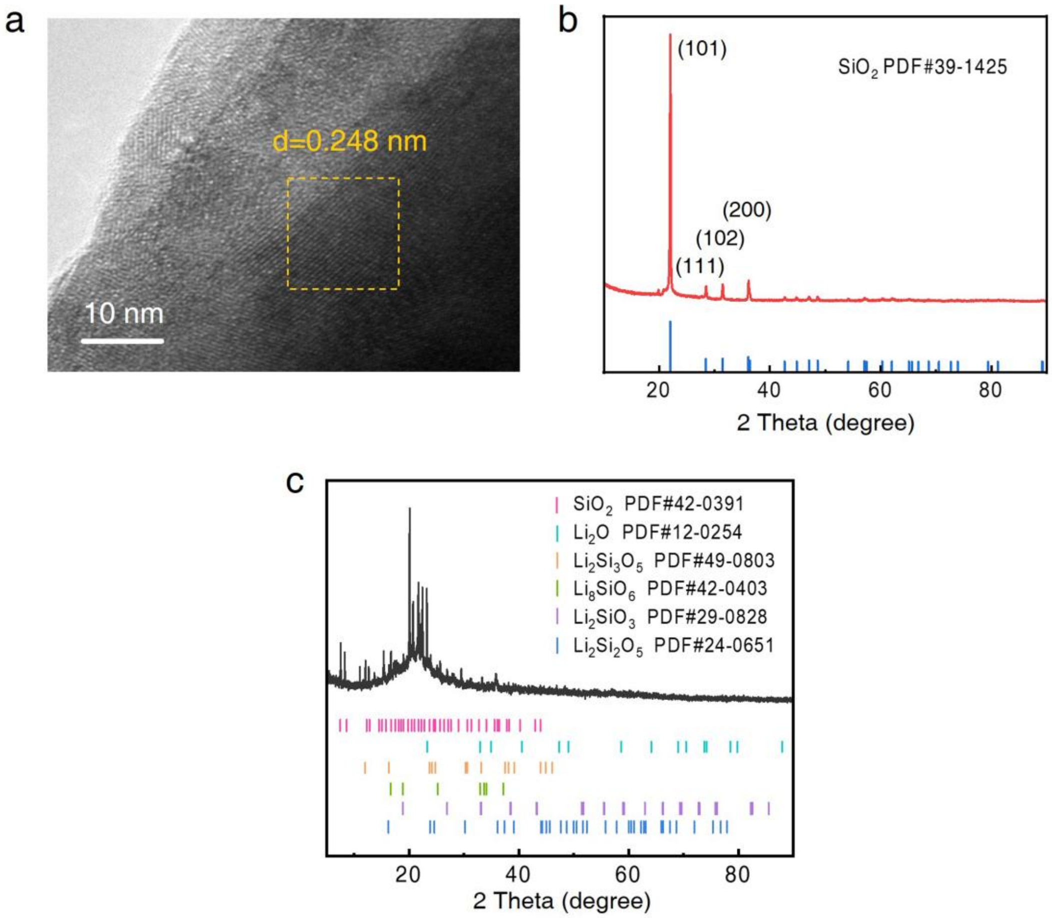

Figure S10. Crystal structure characterization of SiO_x/PCNF before and after lithiation. (**a**) HR-TEM image and (**b**) XRD spectra of SiO_x/PCNF film electrodes. (**c**) XRD spectra of preli-SiO_x/PCNF-3 electrodes.

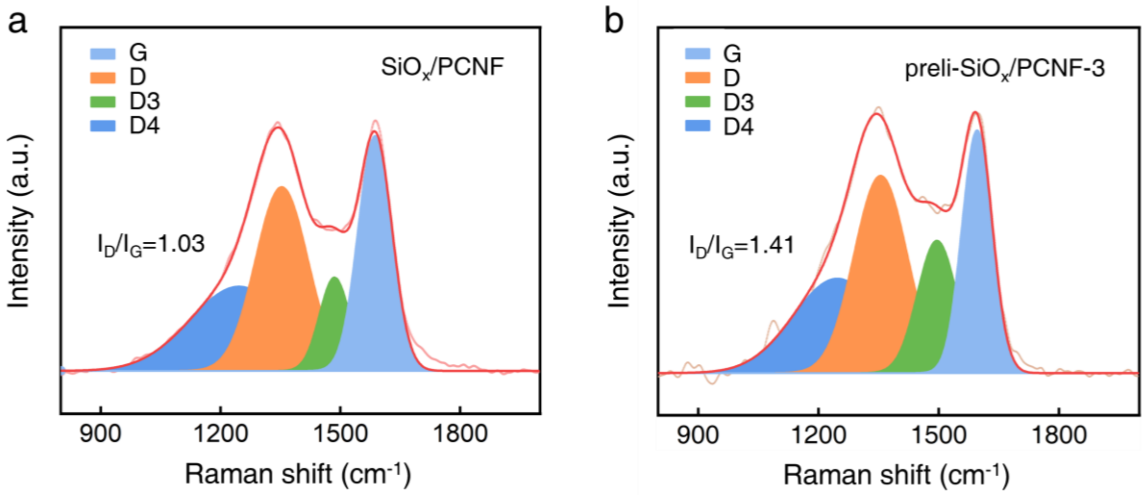

Figure S11. Raman spectra of the (**a**) original SiO_x/PCNF and (**b**) preli-SiO_x/PCNF-3.

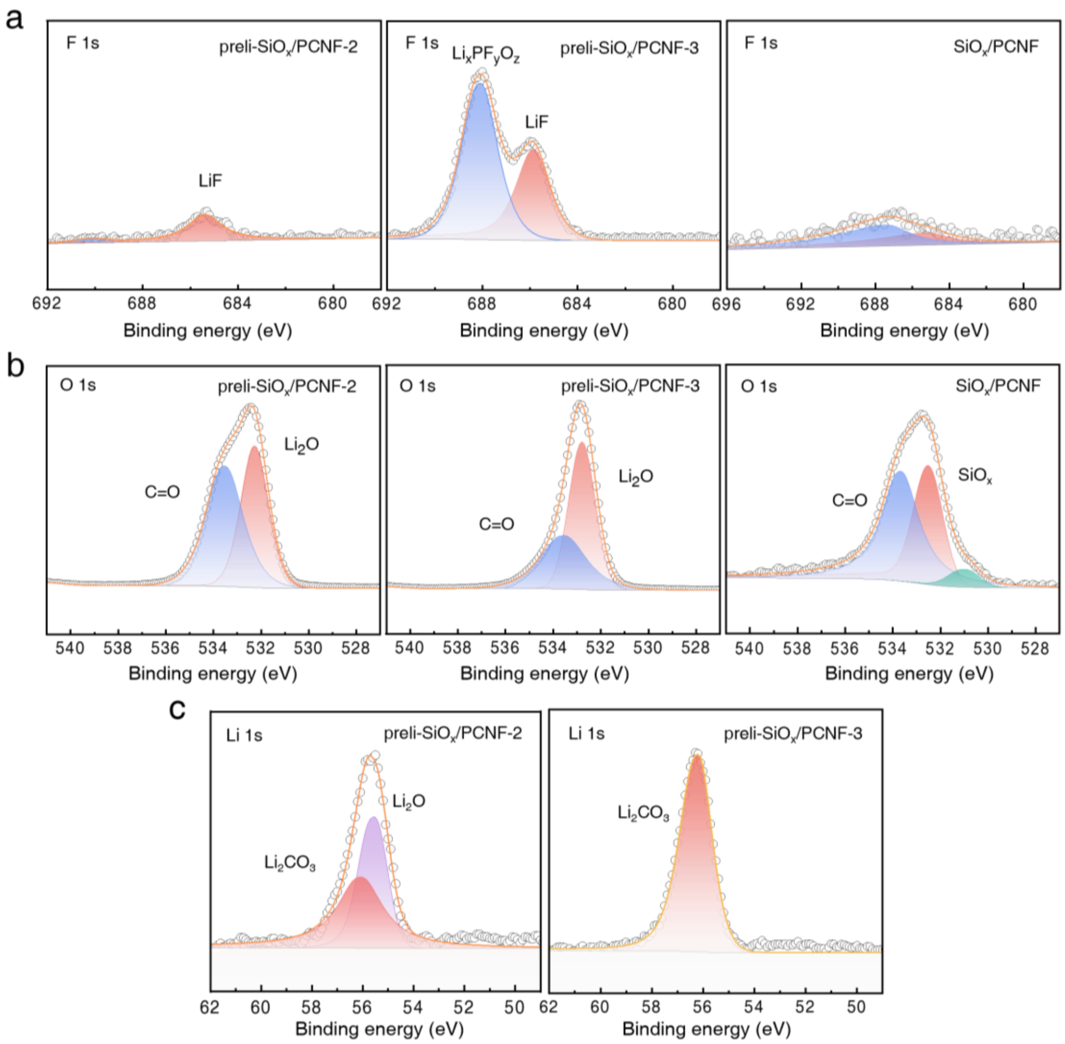

Figure S12. XPS characterization of samples lithiated and unlithiated. XPS spectra of (**a**) F 1s, (**b**) O 1s and (**c**) Li 1s of original electrodes and prelithiated electrodes with Model 2 and Model 3.

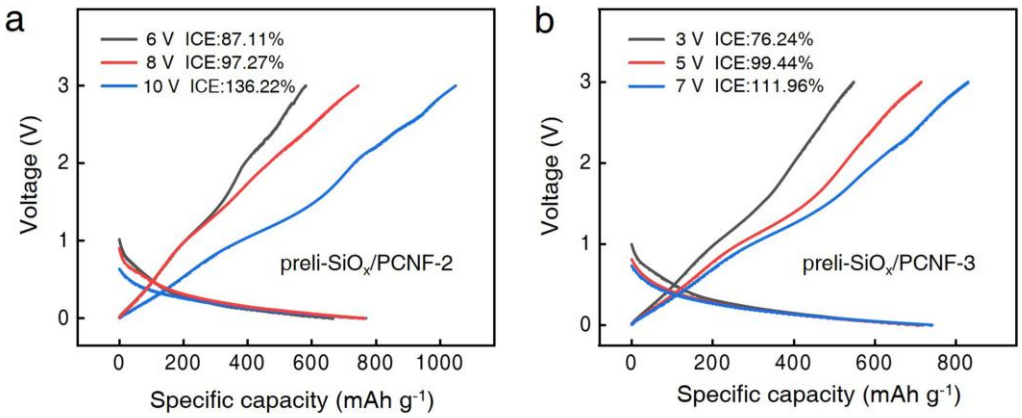

Figure S13. Exploration of optimal conditions for prelithiation models. (**a**) Initial cycle voltage profiles of preli-SiO_x/PCNF-2 after prelithiation at different voltages for 40 s. The results show that 8 V was the optimal voltage. (**b**) Initial cycle voltage profiles of preli-SiO_x/PCNF-3 after prelithiation at different voltages for 30 s. The results showed that 5 V was the optimal voltage.

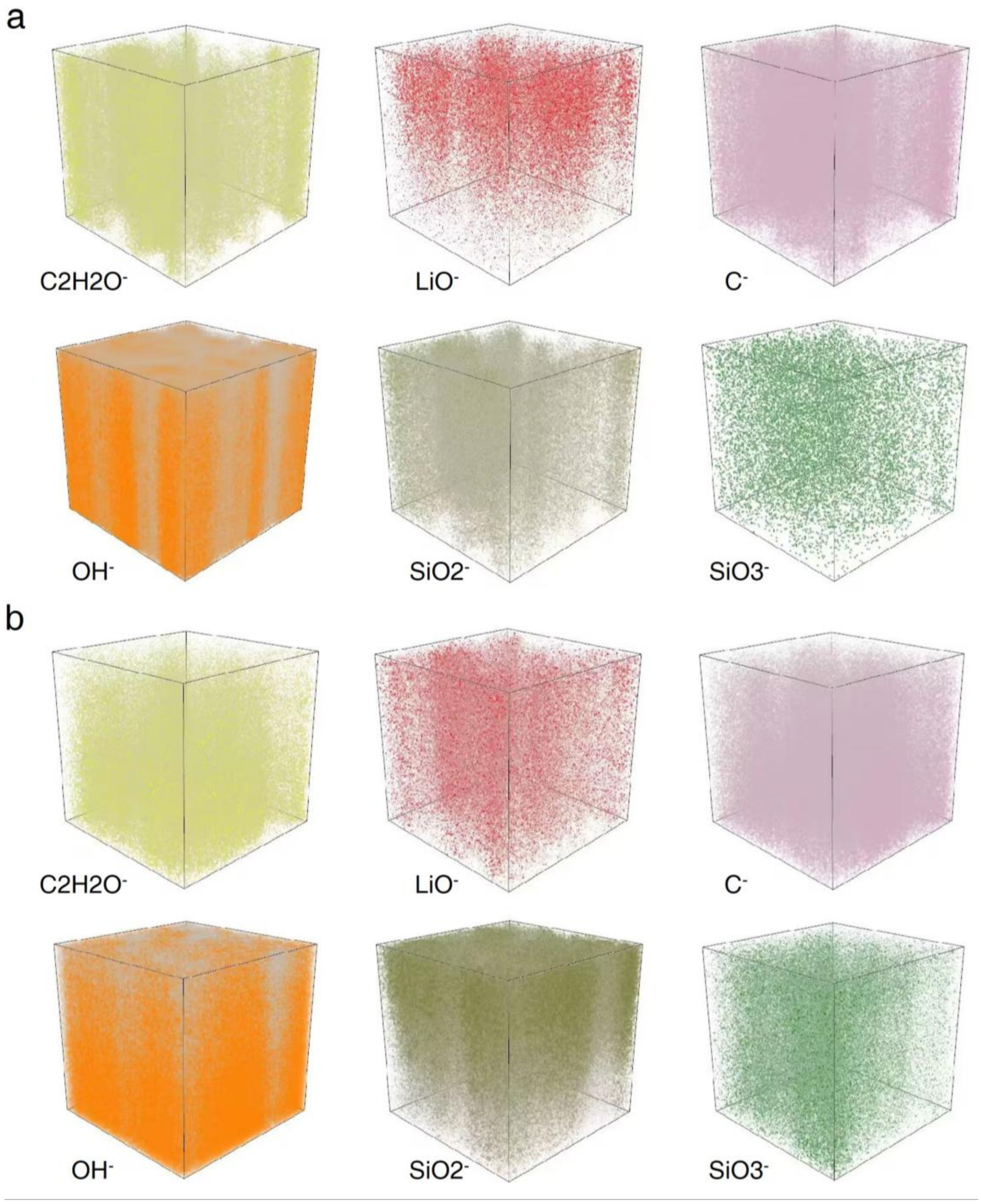

Figure S14. 3D structural views of TOF-SIMs depth sputtering on the surface of (**a**) preli-SiO_x/PCNF-2 and (**b**) preli-SiO_x/PCNF-3. The more saturated the color, the higher the content of the substance.

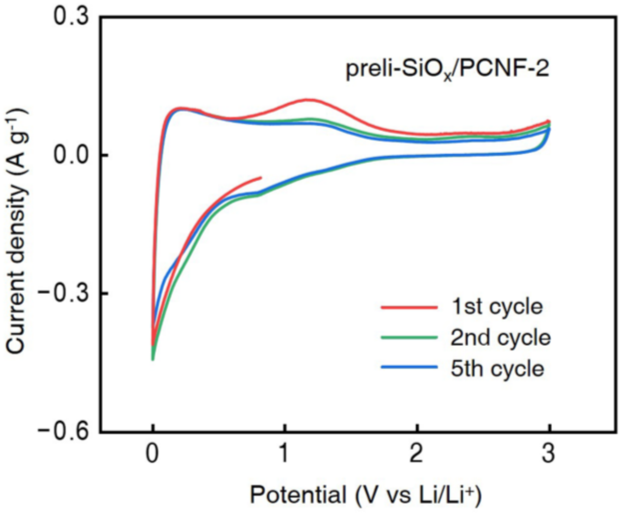

Figure S15. Cyclic voltammetry curves of preli-SiO_x/PCNF-2 anode.

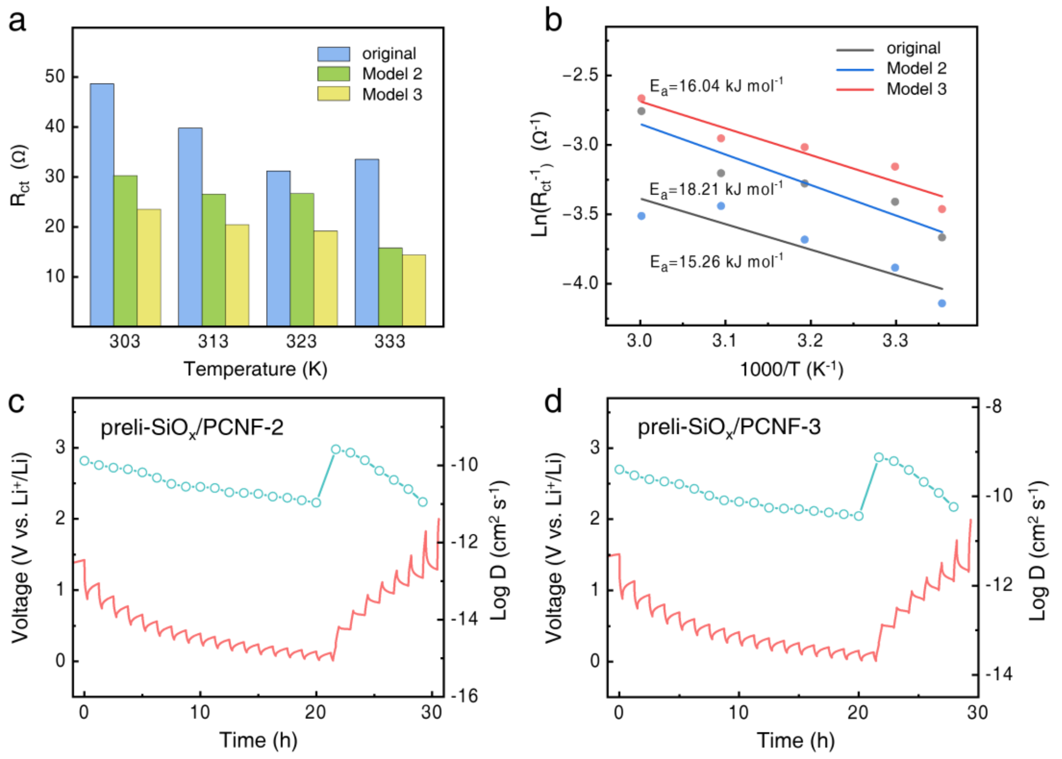

Figure S16. Exploration of the mechanism of Li^+ dynamics. (**a**) Temperature variable (Rct) of Model 2 and Model 3 pre-lithiated anodes. (**b**) Arrhenius desolvation behavior of SiO_x/PCNF, preli-SiO_x/PCNF-2 and preli-SiO_x/PCNF-3 anode in half-cells. (**c-d**) GITT tests of preli-SiO_x/PCNF-2 and preli-SiO_x/PCNF-3 electrodes. From these figures, preli-SiO_x/PCNF-3 had the smallest Rct value among all anodes, indicating that the Li^+ transfer resistance at the interface was the smallest. Rct versus 1/T can reflect the Li^+ diffusion energy barrier at the interface. Preli-SiO_x/PCNF-3 (16.04 kJ-mol^{-1}) had a lower Ea value than that of preli-SiO_x/PCNF-2 (18.21 kJ-mol^{-1}), indicating that the improved interfacial conductive network construction of model 3 resulted in the lowest Li^+ migration resistance. The improved kinetics were also supported by GITT, where the $D_{Li}{}^+$ values of preli-SiO_x/PCNF-3 were relatively higher than those of preli-SiO_x/PCNF-2 for most states of charge (SOC) states, suggesting that it had a faster Li^+ diffusion rate. The smaller Rct value directly reflects the reduction of

interfacial polarization, while the higher D_{Li^+} value in the GITT test indicates that the concentration gradient inside the material was reduced, effectively suppressing the concentration polarization phenomenon. This optimized electrode structure reduces the charge transfer impedance by providing more electron transport channels on the one hand, and shortens the diffusion path of lithium ions by constructing an efficient ion diffusion network on the other.

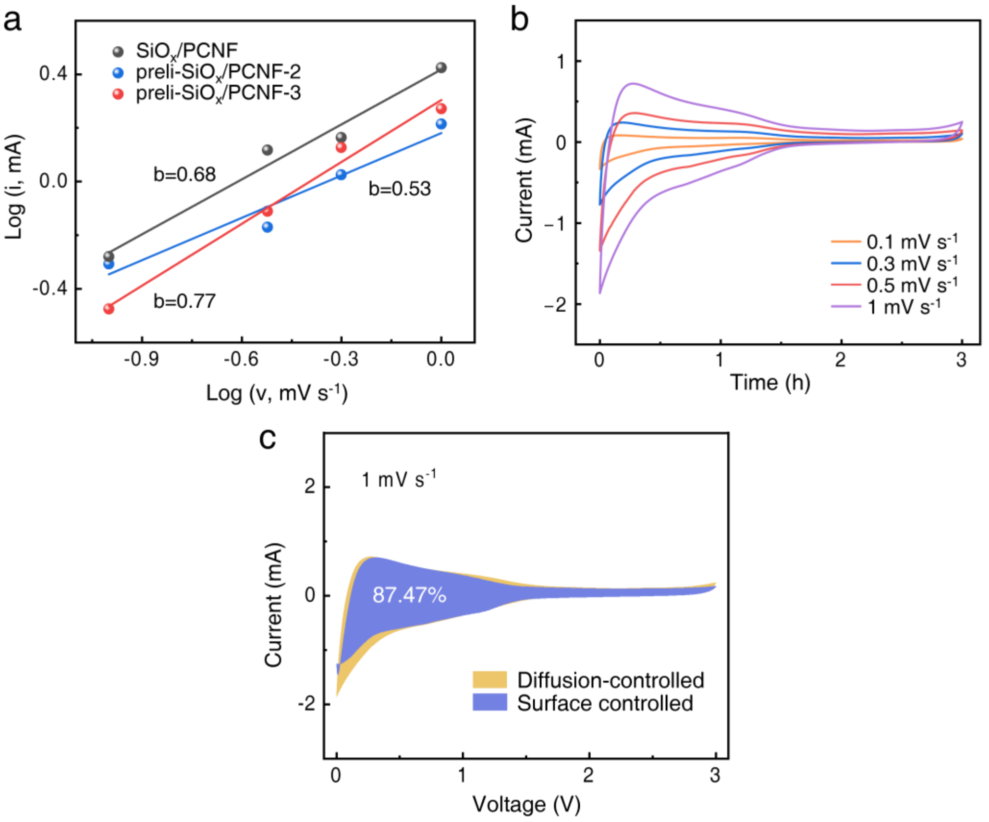

Figure S17. Exploration of Li^+ diffusion mechanisms. (**a**) b-plots of SiO_x/PCNF, preli-SiO_x/PCNF-2 and preli-SiO_x/PCNF-3. (**b**) CV curves of preli-SiO_x/PCNF-3 under various scanning rates. (**c**) Separation of surface-controlled and diffusion-controlled contributions of preli-SiO_x/PCNF-3 at a scan rate of 1 mV s^{-1}. In order to further explore the kinetic mechanism of Li-ion diffusion behavior, we performed CV tests at different scan rates. The total electrochemical capacity can be divided into two parts: surface contribution and diffusion contribution. As shown in Fig. S17a, the b-values of SiO_x/PCNF, preli-SiO_x/PCNF-2, and preli-SiO_x/PCNF-3 are 0.68, 0.53, and 0.77, respectively, with the b-value of preli-SiO_x/PCNF-3 being the closest to 1, which suggests that the electrochemical process prefers the surface control mechanism. Fig. S17b demonstrates the good redox peak morphology of the material maintained at different scan rates, confirming its excellent kinetic responsiveness. Further quantitative analysis shows (Fig. S17c) that the surface-controlled contribution of preli-

SiO_x/PCNF-3 is as high as 87.47% at a scan rate of 1.0 mV s^{-1}, which dominates the process. This hybrid storage mechanism dominated by surface control provides a key microscopic mechanism to support the excellent multiplicity performance and cycling stability exhibited by preli-SiO_x/PCNF-3.

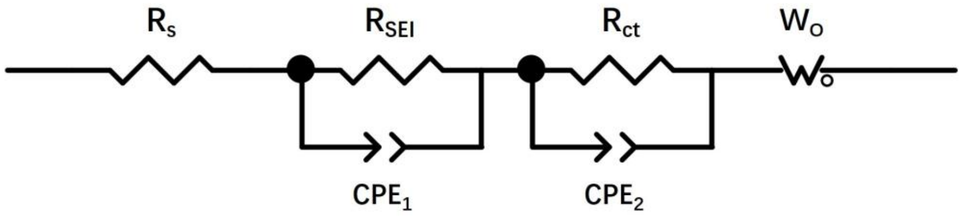

Figure S18. Analog circuit of the Nyquist plots, where R_s denotes the ohmic internal resistance, R_{SEI} denotes the impedance of the SEI membrane, the size of which is related to the number and structural stability of the SEI membrane, R_{ct} denotes the charge transfer impedance, and Warburg impedance W_o describes the ionic diffusion impedance.

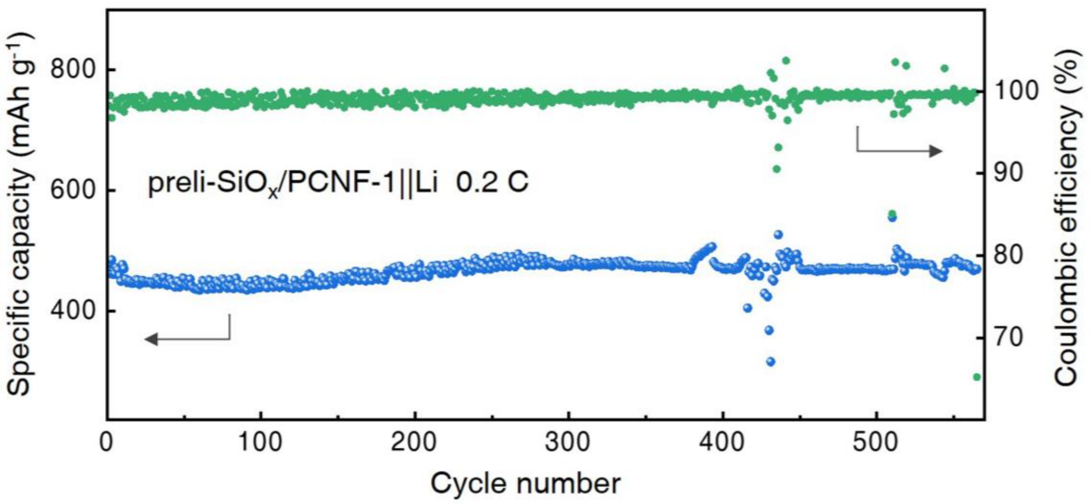

Figure S19. Long cycling performance of preli-SiO_x/PCNF-1||Li at 0.2 C.

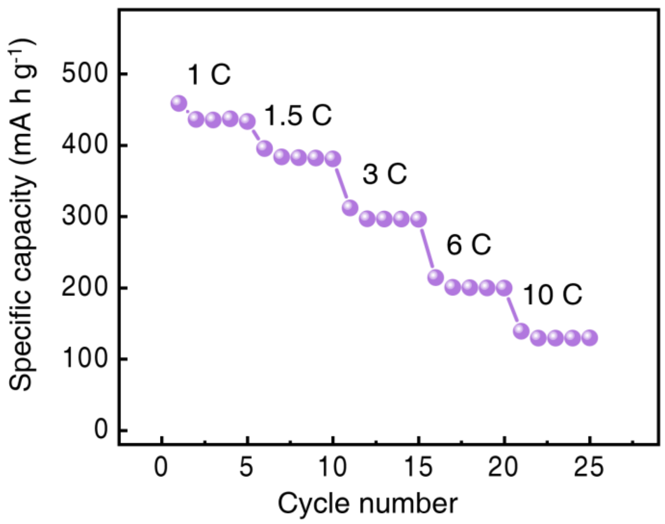

Figure S20. High-rate performance of preli-SiO_x/PCNF-3 anode.

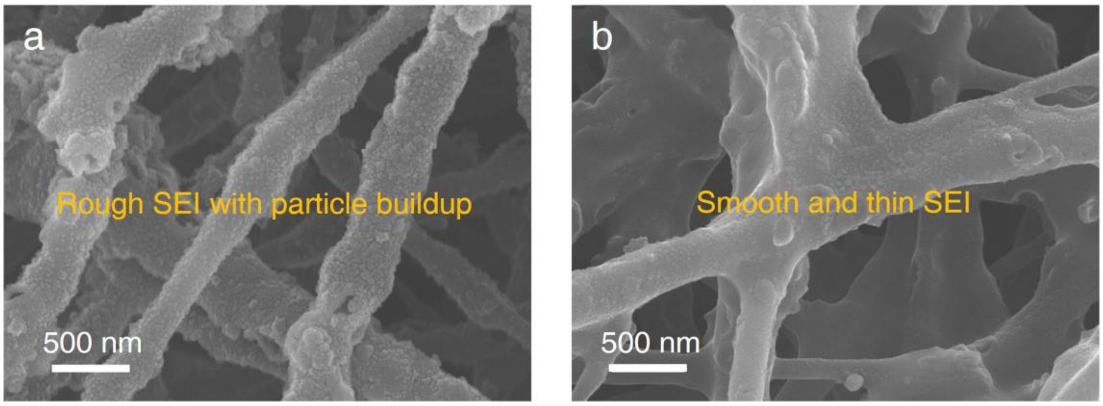

Figure S21. SEM images of prelithiated SiO_x/PCNF membranes with (**a**) Model 2 and (**b**) Model 3 after 1 cycle.

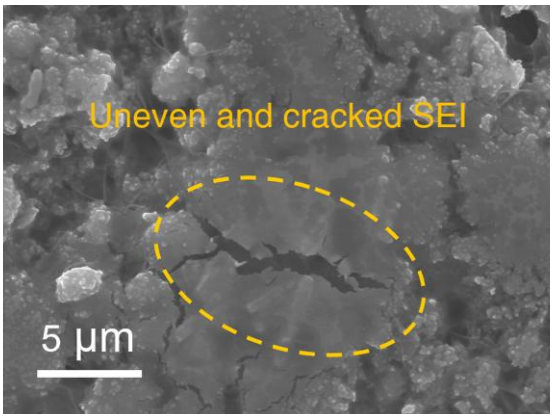

Figure S22. SEM images of unprelithiated SiO_x/PCNF membrane after 500 cycles.

Supporting Tables

Tab. S1 The resistance values of the different circuit elements were fitted using Z-View software.

**Sample**	**Condition**	**R_s (Ω)**	**R_{SEI} (Ω)**	**R_{ct} (Ω)**
preli-SiOx/PCNF-2	before cycle	0.9177	31.71	5.989
	1st cycle	1.656	25.32	3.717
	10th cycle	1.472	11.95	2.471
	50th cycle	1.368	18.05	23.4
preli-SiO_x/PCNF-3	before cycle	1.108	25.4	6.056
	1st cycle	1.226	22.43	11.27
	10th cycle	1.42	12.8	5.099
	50th cycle	1.982	9.031	6.39

Tab. S2 Equivalent circuit of the SiO_x/PCNF, preli-SiO_x/PCNF-2 and preli-SiO_x/PCNF-3 half-battery after 1000 cycles.

**Sample**	**R_s (Ω)**	**R_{SEI} (Ω)**	**R_{ct} (Ω)**
SiO_x/PCNF	76.05	348.2	248.9
preli-SiO_x/PCNF-2	27.34	197.5	150
preli-SiO_x/PCNF-3	23.34	142.4	188.4

Tab. S3 Performance comparison of prelithiation strategy between published literature and this work.

Method	Time	ICE (before lithiation)	ICE (after lithiation)	ICE promotion rate	Materials	Reference
Vacuum thermal evaporation	180 s	74.0%	91.0%	22.97%	C	[19]
Chemical prelithiation	2 min	88%	98%	11.36%	SiO_x/C	[37]
Direct contact prelithiation	24 h	72.5	96.9%	33.66%	Si	[38]
Chemical prelithiation	4 min	75.5%	99.5%	31.79%	HC	[39]
Electrochemical prelithiation	30 min	73.6%	94.9%	28.94%	SiO_x/C	[40]
Direct contact prelithiation	24 h	53.2%	90.0%	69.17%	C	[41]
Electrochemical prelithiation	30 s	57.9%	99.44%	71.74%	SiO_x/C	This work
